# The trajectories of cardiometabolic multimorbidity associate with mortality risk: a study based on Guangdong adult chronic diseases and risk factors cohort

**DOI:** 10.3389/fpubh.2026.1833156

**Published:** 2026-07-09

**Authors:** Shanghui Ye, Xiuling Song, Maigeng Zhou, Zhenping Zhao, Yuchang Zhou, Xuan Yang, Jianxiong Hu, Keqing Liang, Aga Zheng, Ni Xiao, Liming Pu, Xueyan Zheng, Ye Wang, Dejian Zhao, Yu Liao, Tao Liu, Ruilin Meng, Wenjun Ma, Guanhao He

**Affiliations:** 1 Department of Public Health and Preventive Medicine, School of Medicine, Jinan University, Guangzhou, China; 2 Guangdong Provincial Center for Disease Control and Prevention, Guangzhou, China; 3 The National Center for Chronic and Noncommunicable Disease Control and Prevention, Beijing, China

**Keywords:** cardiometabolic multimorbidity, chornic disease, cohort study, disease trajectories, mortality

## Abstract

**Background:**

Cardiometabolic multimorbidity (CMM) prevalence has risen, but longitudinal evidence on trajectories and mortality links remains limited.

**Objective:**

This study aimed to examine CMM trends and the impact of its statuses/trajectories on mortality in Guangdong, China.

**Design:**

This is a cohort study.

**Setting:**

Data were collected from the Guangdong Provincial adult chronic diseases and risk factors cohort (GDACRC). The follow-up data were collected from the hospitalization record system and the vital register system in Guangdong Province.

**Participants:**

Baseline surveys were separately conducted in 2007, 2010, 2013, 2015, and 2018, which recruited 34,244 participants aged 18 years or older.

**Measurements:**

Calculated age-sex standardized CMM prevalence. Cox proportional hazards models were used to assess the associations between CMM statuses/trajectories and mortality risk.

**Results:**

From 2007 to 2018, the prevalence of CMM increased significantly from 4.31 to 8.75% in Guangdong Province, China (*p* < 0.05). Compared with participants without any cardiometabolic diseases (CMD), the mortality risk of those with single CMD and CMM, respectively, increased by 30 and 85%, with a greater risk of patients aged < 65 years. The most common trajectory was from metabolic disease (M) to cardiovascular diseases (C), that 9.31% healthy participants at baseline developed CMM on this trajectory, which accounted for 53.5% of all CMM patients. Compared to patients with single M, patients with the trajectory from C to M had greater mortality risk (HR = 3.32, 95% CI: 2.13–5.16).

**Limitation:**

Single-province sample; self-report bias.

**Conclusion:**

CMM represents a major public health challenge in Guangdong Province, China. Our findings provide new insights on the CMM progression, which may be helpful for prevention and clinical management of CMM.

## Introduction

Multimorbidity is generally understood to be the presence of two or more chronic conditions (1). Among various multimorbidity patterns of chronic diseases, cardiometabolic multimorbidity (CMM) is one of the main patterns. CMM is defined as the co-occurrence of at least two cardiometabolic diseases (CMDs), including type 2 diabetes (T2D), coronary heart disease (CHD), stroke, and hypertension (2, 3). These four diseases exhibit strong biological interconnections through shared etiological pathways, including insulin resistance, endothelial dysfunction, chronic inflammation, and neurohormonal activation, that promote their clustering and mutual progression (4). Epidemiological studies have reported a substantial burden of cardiometabolic multimorbidity (CMM) across various populations. For example, in South Africa, the prevalence of CMM was 25.87% among adults aged ≥45 years (5), while in China it reached 30.6% among those aged ≥64 years (6). Similarly, data from Mexico indicate that 44.5% of individuals aged ≥60 years have at least two cardiometabolic conditions (7).

Accelerated aging exacerbates the progression of multimorbidity of CMM. With the proportion of older adults (aged 65 and over) in the United States rising from 12.4 to 16.8% during 2000–2020 (8), the CMM prevalence has increased from 9.4 to 14.4% among American adults in parallel (9). An epidemiological study also demonstrated that the risk of developing CMM rises progressively with age: the relative risk increased from 1.94 in adults aged 50–54 years to 6.79 in older adults aged 70–74 years (10). In recent years, population aging in China has accelerated markedly from 8.87% in 2010 to 13.50% in 2020 (11), portending a substantial burden of CMM. As China’s largest provincial economy, Guangdong has retained a younger population owing to the massive inflows of young migrant workers from inland provinces. Nevertheless, its two decades of rapid economic transition and urbanization have reshaped residents’ lifestyles and chronic disease risk factors at an earlier stage compared with other Chinese provinces (12). Even with a lower aging rate, these changes make Guangdong a “leading indicator” for CMM trends that will likely emerge nationwide in the coming years (13). Moreover, the province hosts the Guangdong Provincial Adult Chronic Diseases and Risk Factors Cohort (GDACRC), a provincially representative, rigorously designed cohort based on stratified multistage cluster sampling. This ensures robust, generalizable data to investigate CMM epidemiology across diverse subpopulations. Thus, Guangdong serves as a study setting to generate up-to-date evidence on CMM, providing insights that inform both targeted local interventions and national public health strategies.

CMM significantly elevates risks of adverse health outcomes, including disability, hospitalization, and premature mortality. For instance, evidence from a Swedish study found older adults with CMM were more likely to experience physical weakness (RR = 2.25, 95%CI: 1.13–4.49) (14). The patients with CMM also had greater risk of disability (OR = 3.0; 95% CI 1.6–5.6) (15). CMM has caused great health burden in China. According to a systematic analysis of Global Burden Disease, stroke, ischemic heart disease, hypertensive heart disease, and diabetes mellitus ranked 1st, 2nd, 10th, and 20th in cause-specific years of life lost (YLLs) among 282 causes of death (16), respectively.

Previous evidence has also shown that different trajectories of the same multimorbidity have varying impacts on health outcomes. For instance, a longitudinal study of 1.7 million Welsh participants revealed that individuals experiencing the consecutive onset of diabetes, psychiatric disorders, and congestive heart failure demonstrated the most pronounced reduction in life expectancy (13.23 years), whereas alternative temporal sequences of these multimorbidity were associated with comparatively smaller reductions (17). The Academy of Medical Sciences has emphasized that longitudinal trajectory-based approaches can enhance understanding of disease progression and the patterns of disease clustering over time and has proposed the concept of “multimorbidity trajectories” (18). Importantly, different sequences of disease accumulation may reflect heterogeneous underlying mechanisms and lead to substantially different prognostic outcomes, even when the final disease count is similar. However, there is limited research on the development trajectory of CMM and its association with mortality, particularly in terms of identifying high-risk progression pathways and critical windows for early intervention, which hampers a more comprehensive understanding of high-risk trajectories of CMM for prevention and clinical management.

To address the knowledge gaps, our study collected data from the Guangdong Provincial Adult Chronic Diseases and Risk Factors Cohort (GDACRC). We first described the temporal trend of age-sex standardized CMM prevalence from 2007 to 2018 and then investigated the associations between both CMM and the trajectory of CMM with mortality. Our findings will provide evidence-based information to prevent CMM and manage patients with CMM.

## Materials and methods

### Study design

This study is based on Guangdong Provincial Adult Chronic Diseases and Risk Factors Cohort (GDACRC). The baseline surveys were separately conducted in 2007, 2010, 2013, 2015, and 2018, which included 34,244 participants. Each survey round enrolled an independent sample, and no participant was included in more than one wave. The follow-up data on incidence and mortality of CMM were collected from the hospitalization record system and the vital registry system in Guangdong (Supplementary Figure S1). The age-sex standardized CMM prevalence in Guangdong Province during study period was calculated based on the baseline survey data, and the association of the trajectory of CMM with mortality was estimated by Cox proportional hazards model. Segmented model outputs indicated that hazard ratios for both CMM status and trajectory groups were stable within the first 12 years of follow-up but became increasingly variable thereafter. Therefore, the primary analysis was restricted to the 0-to-12-year follow-up window.

#### Data collection at baseline

A total of 30 surveillance sites (counties/districts) were randomly selected in Guangdong Province. Adults aged over 18 years old living in the study sites for at least 6 months were recruited. All participants were interviewed face-to-face using a structured questionnaire by well-trained public health practitioners from the local centers for disease control and prevention or community health service centers. The questionnaire included status of chronic diseases, demographic information, and lifestyle. The common chronic diseases included hypertension, type 2 diabetes mellitus (T2D), myocardial infraction, atrial fibrillation, angina pectoris, stroke, chronic obstructive pulmonary disease, asthma, cancer, chronic digestive system diseases, and chronic urinary system diseases. Participants with missing baseline data on hypertension, T2D, CHD, or stroke status were excluded from the analysis.

Socio-demographic characteristics and lifestyles were collected via a questionnaire. Socio-demographic characteristics included education level (primary school and below, middle school, or bachelor’s degree and above), marital status (married, never married, divorced, or widowed), annual income level (low-income and high-income), and residential area (urban or rural). Lifestyle factors comprised smoking status (current/former smokers or never smokers), alcohol consumption (current/former drinkers or never drinkers), physical activity (<150 min of moderate-intensity activity per week, and ≥ 150 min of moderate-intensity activity per week), sedentary behavior (≥8 h daily non-sleep sedentary time and < 8 h daily non-sleep sedentary time), and sleep duration (≥7 h per day, and < 7 h per day). In addition, some physical examinations including height, weight, waist circumference, and blood pressure were also collected through standardized methods, which have been described in previous studies (19, 20). All participants were provided written informed consent. The inclusion and exclusion criteria of participants have been reported previously (21).

#### Data collection at follow-up

The incidence of CMM at follow-ups was extracted from the medical record front page in the clinical database of Guangdong Province from 2012 to 2023. The dataset included patient’s clinical information such as admission/discharge time, diagnosed diseases, and corresponding Classification of Diseases 10th Revision (ICD-10) codes. The dataset was matched with baseline survey data based on each patient’s ID number.

The death records were sourced from the vital registration system of Guangdong Province between June 2009 and April 2024, including the time of death and the cause of death. Death data were also linked with baseline survey data based on the ID number.

**Figure 1:**
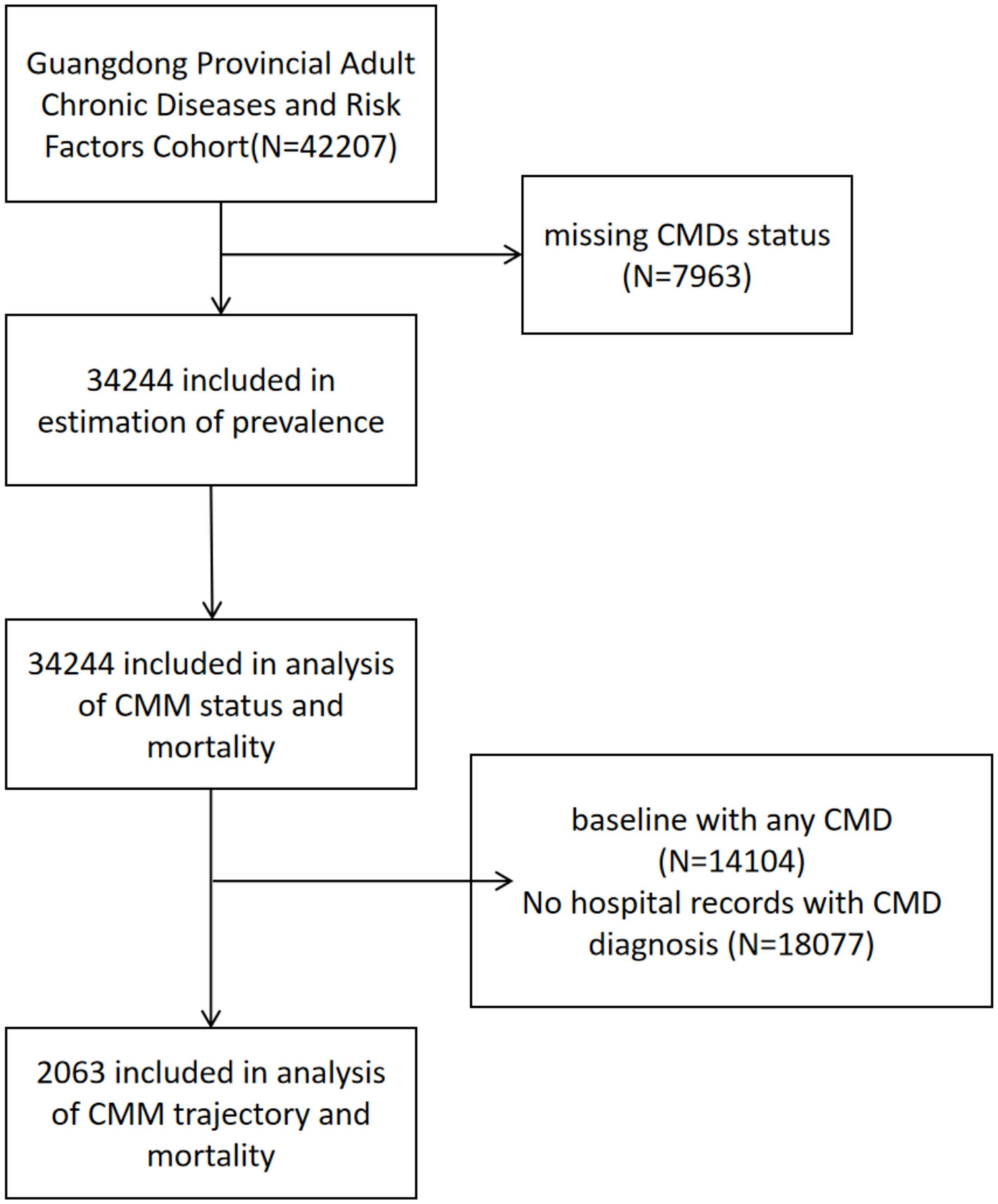
Flowchart of the study. CMDs, cardiometabolic diseases; CMM, cardiometabolic multimorbidity.

Data linkages across baseline surveys, hospitalization records, and death registry were performed via deterministic matching using the ID number as the unique identifier (Figure 1).

#### CMM definition

As stated in the Introduction, T2D, CHD, stroke, and hypertension exhibit strong biological interconnections via shared pathways (e.g., insulin resistance, endothelial dysfunction, and chronic inflammation), and this disease set has been consistently used to define CMM in large-scale epidemiological studies; therefore, CMM was defined as the co-occurrence of at least two of the four CMDs: T2D, CHD, stroke, and hypertension. In the baseline survey, T2D was diagnosed as fasting blood glucose ≥7 mmol/L or self-reported T2D diagnosed by physician diagnosis. Hypertension was defined as systolic blood pressure ≥140 mmHg or diastolic blood pressure ≥90 mmHg, or self-reported hypertension diagnosed by physician. CHD and stroke are also self-reported. During follow-ups, the incidence of CMD was collected from medical record front page. CHD included ischemic heart disease (ICD code: I20–I25) and atrial fibrillation (ICD code: I48); stroke encompassed subarachnoid hemorrhage, intracerebral hemorrhage, other non-traumatic intracranial hemorrhage, cerebral infarction, and stroke (ICD code: I60-I64); hypertension was classified under I10–I15, and T2D corresponded to E11.

### Statistical analysis

#### Estimation of age-sex standardized CMM prevalence

Sample characteristics at baseline were described according to participants’ CMD disease status (Table 1). Then, by merging samples from five rounds of baseline survey, we calculated age-gender standardized weight using the following Equation 1:


$$w_{\mathrm{ij}}=\frac{N_{\mathrm{ij}}}{\sum_{i=1}^{2}\sum_{j=1}^{2}N_{\mathrm{ij}}}$$ (1)

where $N_{\mathrm{ij}}$ denotes the proportion of the standard population in the i age stratum and j sex stratum. Ages were divided into two groups: under 65 years and ≥65 years, and sex included male and female. Age-sex standardized prevalence was calculated using direct standardization to the pooled study population across all survey waves (2007, 2010, 2013, 2015, and 2018). For each survey round of the year *t,* the adjusted prevalence $P_{t}^{\mathrm{adj}}$ was derived by the following Equation 2:


$$P_{t}^{\mathrm{adj}}=\sum_{i=1}^{2}\sum_{j=1}^{2}(w_{\mathrm{ij}}.\frac{C_{t,\mathrm{ij}}}{N_{t,\mathrm{ij}}})\times 100\%$$ (2)

where crude prevalence was derived as $C_{t}$ / $N_{t}\times 100\%$ , with $C_{t}$ and N_t_ representing year-specific case counts and survey sample size, respectively.

**Table 1:** Baseline characteristics of participants without CMD, with single CMD, CMM in Guangdong provincial adult chronic diseases and risk factors cohort (*N* = 34,244).

Characteristics	Without CMD (*N* = 21,262)	With single CMD (*N* = 10,633)	CMM (*N* = 2,349)
Age			
<65	19,061 (89.65%)	7,503 (70.56%)	1,382 (58.83%)
≥65	2,201 (10.35%)	3,130 (29.44%)	967 (41.17%)
Sex			
Male	9,171 (43.13%)	4,927 (46.34%)	1,091 (46.45%)
Female	12,091 (56.87%)	5,706 (53.66%)	1,258 (53.55%)
Resident			
Urban	11,755 (55.29%)	5,582 (52.50%)	1,275 (54.28%)
Rural	9,507 (44.71%)	5,051 (47.50%)	1,084 (45.72%)
Region			
GBA	8,317 (39.12%)	3,606 (33.91%)	841 (35.80%)
Non-GBA	12,945 (60.88%)	7,027 (66.09%)	1,508 (64.20%)
Education			
Primary school and below	4,685 (22.03%)	3,913 (36.80%)	982 (41.81%)
Primary school	4,108 (19.32%)	2,338 (21.99%)	471 (20.01%)
Junior high school	6,609 (31.08%)	2,597 (24.42%)	523 (22.22%)
High school	3,603 (16.95%)	1,326 (12.47%)	272 (11.58%)
College degree or above	2,257 (10.62%)	461 (4.34%)	101 (4.30%)
Marital status			
Unmarried	1,757 (8.26%)	279 (2.62%)	39 (1.66%)
Married	14,996 (70.53%)	7,970 (74.96%)	1,860 (79.18%)
Divorced	338 (1.59%)	203 (1.91%)	36 (1.53%)
Bereaved	4,171 (19.62%)	2,181 (20.51%)	414 (17.58%)
Smoking status			
Previously smoked	14,939 (70.26%)	7,055 (66.35%)	1,533 (65.26%)
Never smoke	6,323 (29.74%)	3,578 (33.65%)	816 (34.74%)
Drinking status			
Previously drank	12,974 (61.02%)	6,963 (65.48%)	1,685 (71.73%)
Never drink	8,289 (38.98%)	3,670 (34.52%)	664 (28.27%)

The prevalence was defined as the proportion of patients relative to the surveyed population in the survey year, and annual prevalence rates were further calculated according to the number of CMDs (1 CMD, CMM with 2 diseases, and CMM with 3 diseases) during 2007–2018. To evaluate temporal trends of CMM prevalence, linear regression models were applied, with survey year as the independent variable and prevalence as the dependent variable, and the regression coefficient (*β*) and corresponding *p*-value were used to assess the statistical significance and direction of temporal trends. The prevalence trend of different CMMs (e.g., diabetes–hypertension and diabetes–coronary heart disease) was also estimated.

#### Estimation of association between CMM and mortality

Based on CMM disease status in the baseline survey and hospitalization records, the cohort was categorized into three groups: (1) without CMD, (2) single CMD, and (3) cardiometabolic multimorbidity (CMM). For the group without CMD, the time origin was defined as the date of the baseline survey since these participants never developed any CMD during the entire follow-up. For the single CMD and CMM groups, the time origin was defined as the date on which the respective disease state was first reached, namely, the date of first C or M diagnosis for the single CMD group, and the date of CMM onset for the CMM group. All participants were followed from their group-specific time origin until the earliest of death or the censoring date of 31 December 2024. Using the group without CMD as a reference, multivariable-adjusted Cox proportional hazards models were employed to estimate hazard ratios (HRs) and 95% confidence intervals (CIs) of the associations CMD with mortality. The Cox regression model describes the rate of event occurrence by constructing a risk function as following Equation 3:


$$h(t)=h_{0}(t)\exp(\text{Statu}s_{\mathrm{CMM}}+\beta_{1}C_{1}+\ldots +\beta_{k}C_{k})$$ (3)

where h(t) denotes instantaneous risk at the time *t*. $h_{0}(t)$ is the benchmark risk function, representing the risk when all explanatory variables are 0. $\text{Statu}s_{\mathrm{CMM}}$ refers to the status of CMM. $C_{1}$ , $C_{2}$ ,…, $C_{k}$ refer to the covariates including, age, sex, residence, education level, marital status, occupation, drinking status, smoking status and presence of other chronic diseases in the model, and $\beta_{1}$ , $\beta_{2}$ ,…, $\beta_{k}$ are their regression coefficients.

#### Estimation of the trajectory of CMM and its association with mortality

In this analysis, we just included the participants without any CMD in baseline survey. Based on trajectory changes during follow-ups, participants were categorized into four mutually exclusive groups: (1) healthy-M (M, including hypertension or type 2 diabetes), (2) healthy-C (C, including CHD or stroke) (3) healthy-C-M, and (4) healthy-M-C. The time origin for each trajectory group was defined as the date on which the trajectory-defining state was first reached: for the healthy-M group, the date of first M diagnosis; for the healthy-C group, the date of first C diagnosis; for the healthy-C-M group, the date of CMM onset; and for the healthy-M-C group, the date of CMM onset. If a participant never developed any CMD throughout follow-up, they were not included in this trajectory analysis. All participants were followed from their group-specific time origin until the earliest of death or the administrative censoring date of 31 December 2024. Multivariable Cox proportional hazards regression models were used to estimate hazard ratios (HRs) and 95% confidence intervals (CIs), with the healthy-M group as the reference. Adjustments included covariates described in Equation 3.

#### Subgroup analyses and interaction effects assessment

To evaluate the modification effect of socio-demographic and behavioral factors, we performed several subgroup analyses stratified by sex (male or female), age (<65 or ≥65), region (urban or rural), smoking status (current/smoker or never smoke), and drinking status (current/former drinker or never drink). Additionally, interaction effects were formally assessed by incorporating multiplicative interaction terms into the Cox proportional hazards model to determine whether the associations differed significantly across these subgroups.

### Proportional hazards assumption and sensitivity analysis

The proportional hazards (PH) assumption was tested for Cox models using scaled Schoenfeld residuals. The Cox model using the full follow-up model violated the PH assumption (Supplementary Table S1). To address this, we performed sensitivity analyses by partitioning the follow-up time at different cut points and evaluating the PH assumption within each interval (Supplementary Tables S2, S3). The results showed that when follow-up was restricted to 0–12 years, the PH assumption was satisfied (Supplementary Table S4). Consequently, the primary results of both the CMM–mortality association analysis and the trajectory–mortality association analysis are presented based on follow-up data truncated at 12 years.

To test the robustness of the results, we performed sensitivity analyses by estimating the association between CMM and its trajectories and mortality, with and without adjusting for covariates. Because of various baseline survey (2007–2018), medical record (2012–2023), and death records (2009–2024), we conducted sensitivity analyses excluding baseline survey participants before 2012.

All analyses were conducted using R software version 4.2.1, with a significance level set at a two-sided *p*-value <0.05.

## Result

### Temporal trends in prevalence of CMD and CMM

A total of 34,244 participants were included across five survey rounds (2007, 2010, 2013, 2015, and 2018), with respective sample sizes of 6,361, 2,532, 8,260, 8,536, and 8,555, respectively. The study encompassed five survey waves (2007, 2010, 2013, 2015, and 2018) with respective sample size of 6,361, 2,532, 8,260, 8,536, and 8,555, respectively. As shown in Supplementary Table S5, the mean age and standard deviation of participants from 2007 to 2018 were 43.69 ± 13.38, 49.26 ± 16.15, 53.26 ± 14.32, 51.97 ± 14.9, and 54.23 ± 13.85 years old, respectively. The proportions of males were 47.53%, 44.71%, 42.71, 45.92, and 43.39% of participants from wave 1 to wave 5.

**Figure 2:**
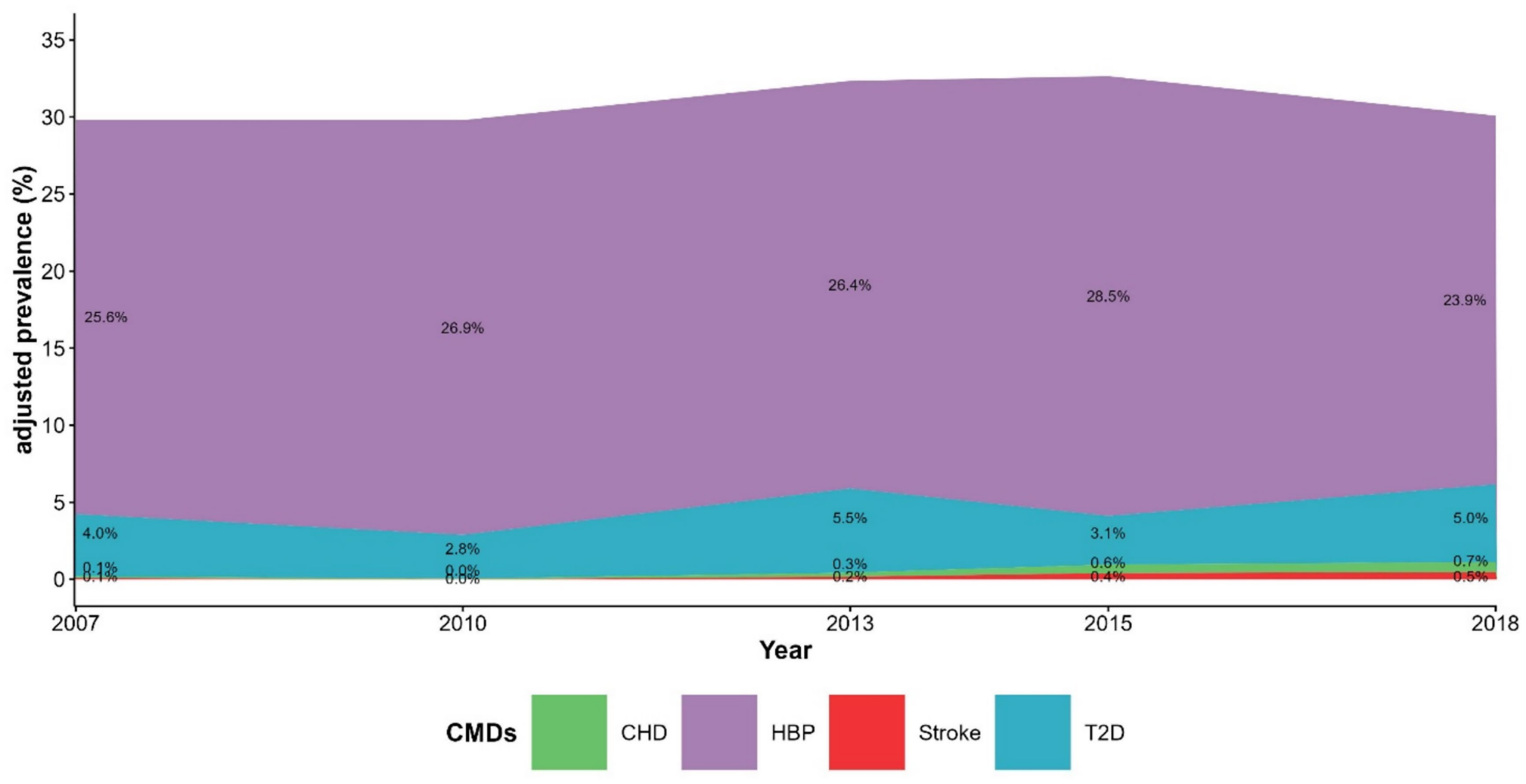
Age-sex standardized prevalence trends of individual cardiometabolic diseases from 2007 to 2018 in Guangdong Province. HBP, hypertension; T2D, type 2 diabetes; CHD, coronary heart disease.

As presented in Figure 2, Supplementary Figure S2, and Supplementary Table S6, the age-sex standardized prevalence of a single CMD in Guangdong Province slightly increased from 29.80% (95%CI: 28.67–30.93%) in 2007 to 30.10% (95%CI: 29.13–31.07%) in 2018, but there is no statistically significant difference (*p* = 0.519). Significant increase was observed in the prevalence of CMM (from 4.31% [95%CI: 3.81–4.81%] to 8.75% [95%CI: 8.15–9.35%]; *p* = 0.021 for trend). Specifically, CMM with 2 diseases increased from 4.12% (95%CI: 3.63–4.61%) to 8.08% (95%CI: 7.50–8.66%) (*p* = 0.036 for trend). Hypertension and T2D multimorbidity were the most common patterns among CMM, rising from 3.68% (95%CI: 3.22–4.14%) in 2007 to 5.97% (95%Cl: 5.47–6.47%) in 2018. CMM with 3 CMDs also had a significant elevation from 0.15% (95%CI: 0.05–0.25%) to 0.63% (95%CI: 0.46–0.80%) (*p* = 0.041). In particular, T2D, hypertension, and CHD multimorbidity were the top pattern among CMM with 3 diseases, with an upward trend from 0.08% (95%CI: 0.01–0.15%) to 0.26% (95%CI: 0.15–0.37%). After stratified analysis, we observed that the prevalence of CMM and its temporal trends was similar between males and females (Supplementary Table S7) and that the prevalence of CMM with 2 and 3 CMDs both had increasing trend. The prevalence of CMM is higher among people aged ≥ 65 years than those < 65 years, but the rate of increase is faster in the younger age group (Supplementary Table S8). Compared to urban regions, rural regions demonstrate a more pronounced increasing trend in CMM prevalence (Supplementary Table S9).

### Associations between CMM and mortality

**Figure 3:**
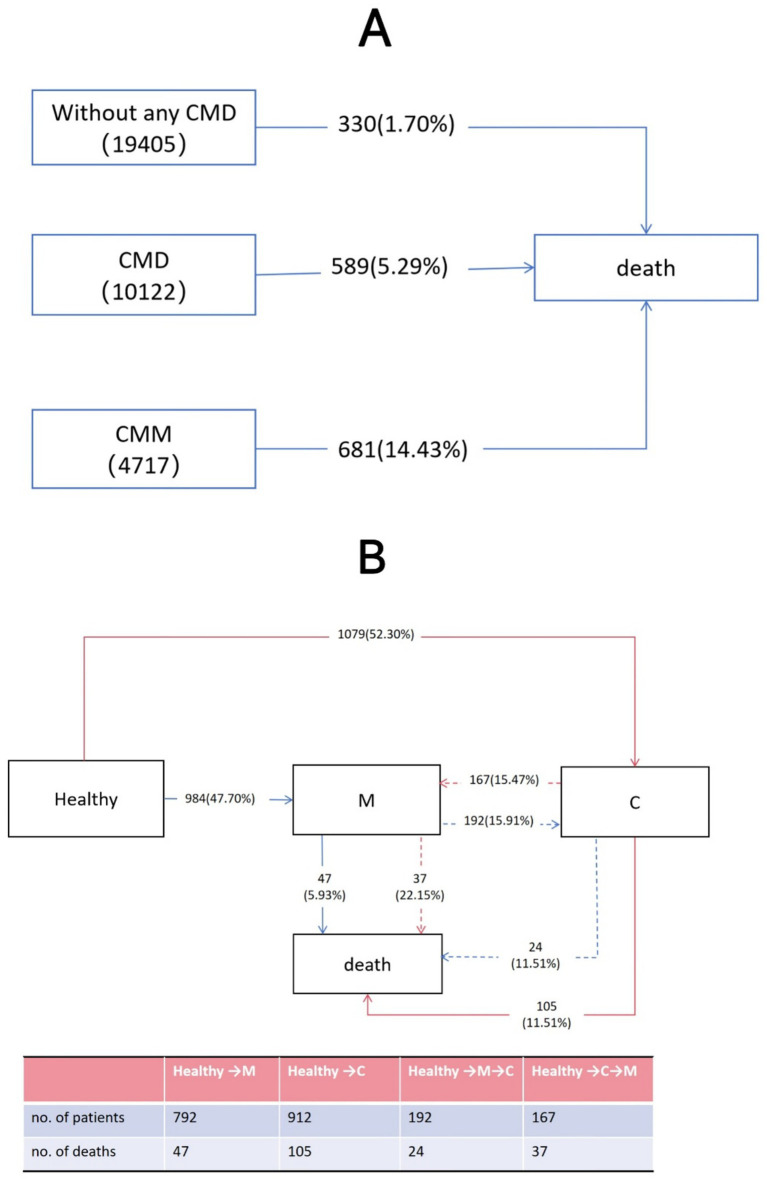
Status transition of CMD. **(A)** The numbers (percentages) of participants from baseline without any CMD, with single CMD, and with CMM to death. **(B)** The numbers (percentages) of each trajectory from baseline without any CMD to death.

Of the 34,244 participants included in this analysis, a total of 19,045 participants remained free of CMDs, 10,122 were diagnosed with single CMDs, and 4,717 had CMM. Among all participants, 1,703 deaths were recorded. During the follow-up, the mortality rates of participants without CMD, with single CMD, and with CMM were 1.70, 5.29, and 14.43%, respectively (Figure 3A).

**Figure 4:**
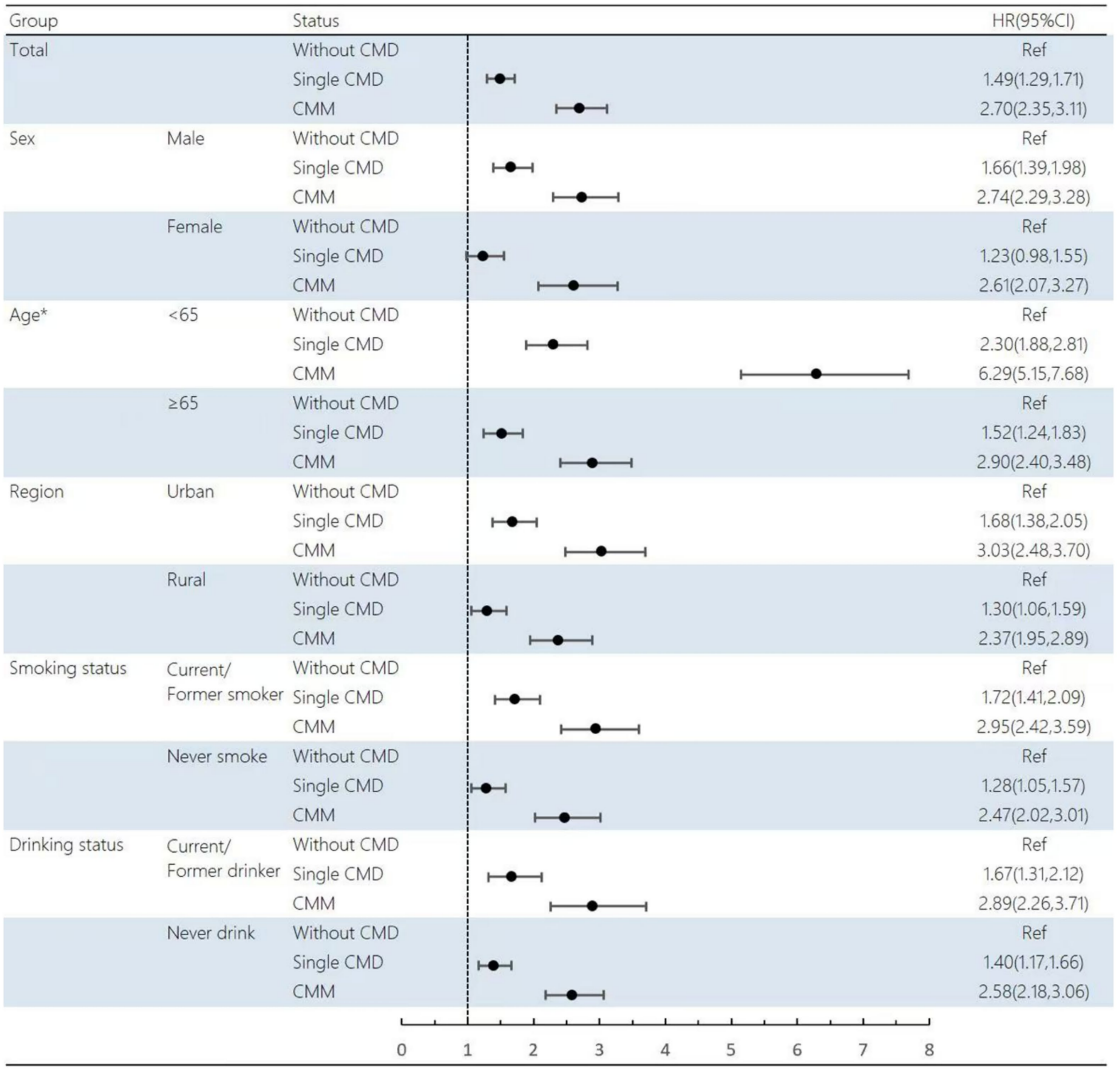
Comparison of hazard ratios (HRs) from health, single CMD, and CMM to death by demographic characteristics and lifestyles. CI, confidence interval; CMD, cardiometabolic diseases (hypertension, coronary heart disease, stroke, and type 2 diabetes). CMM, cardiometabolic multimorbidity: a status with at least two CMDs. *p*-values for interaction were adjusted using Bonferroni correction. *Significant interaction effect at *p* < 0.05.

Compared with participants without CMD, patients with CMM had a significant greater risk of mortality (HR = 2.70, 95% CI: 2.35–3.11) than those with single CMD (HR = 1.49, 95% CI: 1.29–1.71) (*p* < 0.001). Consistent findings were observed across all subgroups (Figure 4; Supplementary Table S10). Moreover, the mortality risk increased with the number of CMD rise [HR from 1.49 (95% CI: 1.29–1.71) for 1 CMD to 3.65 (95% CI: 3.06–4.35) for ≥3 CMDs] (Supplementary Table S11). Furthermore, we observed that mortality risk of CMM in those aged <65 (HR = 6.29, 95% CI: 5.15–7.68) is much higher than those aged ≥65 (HR = 2.90, 95% CI: 2.50–3.48), with significant interaction effect (Figure 4). In addition, the urban residents with CMM (HR = 3.03, 95% CI: 2.48–3.70) had higher mortality risk than rural CMM patients (HR = 2.37, 95% CI: 1.95–2.89) (raw *p* for interaction = 0.065).

### The association between the trajectory of CMM and mortality

**Figure 5:**
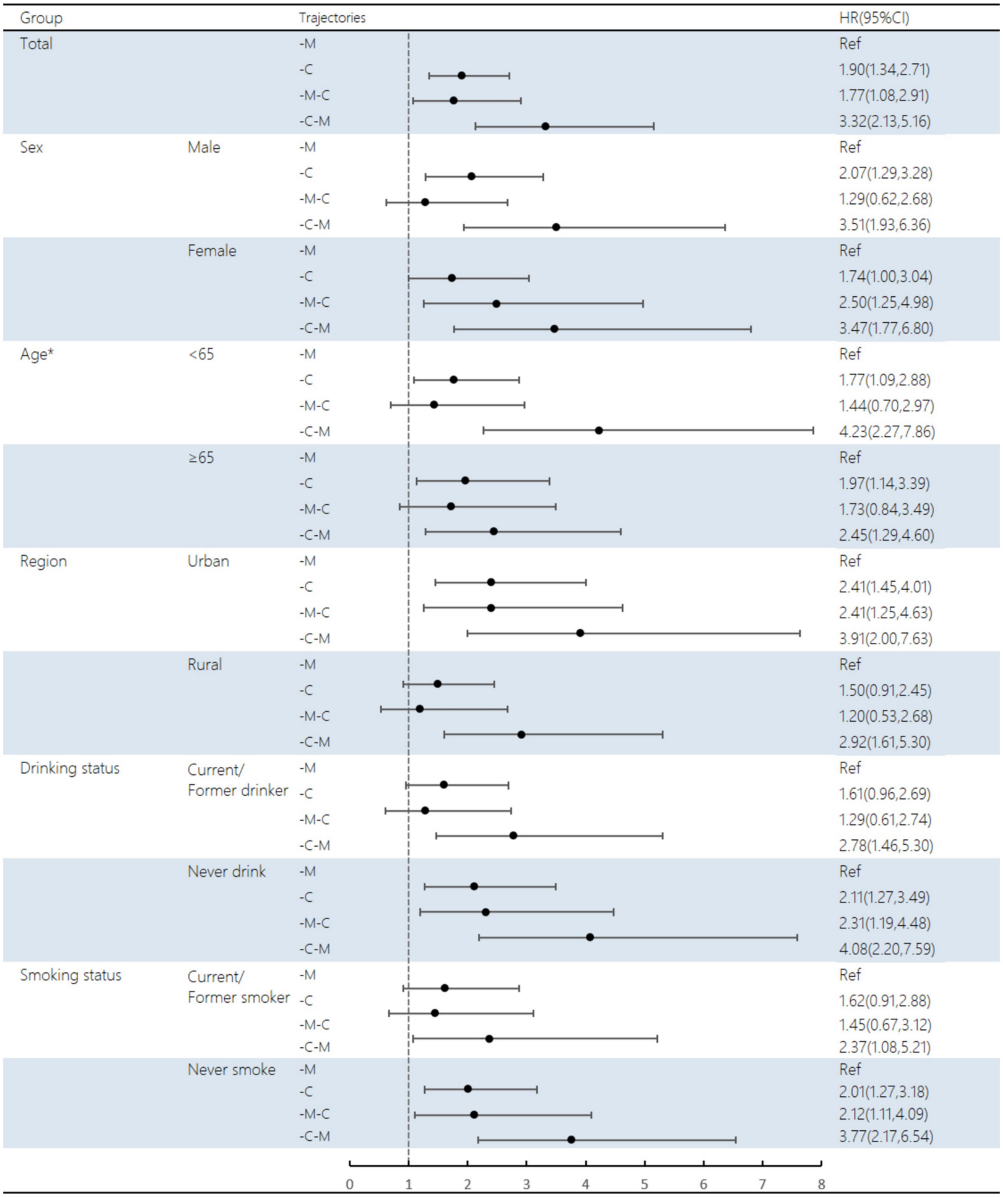
Hazard ratios (HRs) for the multimorbidity development trajectories from baseline to death by demographic characteristics and lifestyle factors. CI, confidence interval; C stands for cardiovascular diseases (coronary heart disease and stroke). M is metabolic diseases (hypertension and type 2 diabetes). *p*-values for interaction were adjusted using Bonferroni correction. *: significant interaction effect at *p* < 0.05.

Of the 2,063 participants included in this analysis, the most common trajectory of CMM was “healthy-M-C,” where 9.31% healthy participants at baseline developed CMM on this trajectory, which accounted for 53.50% of all CMM patients (Figure 3B). Compared to patients with just metabolic diseases, patients with the trajectory of “healthy-C-M” had the highest risk of mortality (HR = 3.32, 95% CI: 2.13–5.16), followed by patients with “healthy-C” trajectory (HR = 1.90, 95% CI: 1.34–2.71) and patients with “healthy-M-C” trajectory (HR = 1.77, 95% CI: 1.08–2.91) (Figure 5). Age-stratified analysis showed that younger adults had the higher mortality risk associated with the “healthy–C–M” trajectory (aged <65 years: HR = 4.23, 95% CI: 2.27–7.86) than older adults (aged ≥65 years: HR = 2.45, 95% CI: 1.29–4.60). There were no significant mortality differences across other stratified groups.

### Sensitivity analysis

We conducted several sensitivity analyses to assess the robustness of our findings through adjustment for covariates in Cox proportional hazards models. The association between multimorbidity status or multimorbidity trajectories and mortality remained stable (Supplementary Tables S6, S8). Considering the discrepancy in time periods between hospitalization records (available since 2012) and death records (available since 2009), we further conducted a sensitivity analysis by excluding data collected before 2012. Furthermore, we exclude participants whose first cardiovascular disease (C) and first metabolic disease (M) were diagnosed within 30 days of each other. E-values were calculated to assess the potential impact of unmeasured confounding on the observed associations (Supplementary Table S11). Overall, the E-value for the association between CMM status and all-cause mortality was 5.05 for the point estimate and 4.34 for the lower confidence limit. This indicates that any unmeasured confounder would need to be associated with both CMM and mortality by a risk ratio of at least approximately 4.34 to shift the lower confidence limit to the null. For the trajectory groups, the E-values for the lower confidence limits ranged from 2.03 to 4.02.

## Discussion

In this cohort study, we observed an increasing age-sex standardized prevalence of CMM in Guangdong Province, China, from 2007 to 2018. We further demonstrated that CMM was associated with higher mortality risk, and those with the trajectory of healthy-C-M linked with the highest risk. These findings provide new insights into the progression trajectory of CMM and its impact on health outcomes, which may inform future CMM prevention and clinical management in China.

We observed that the CMM prevalence was 8.75% in 2018 in Guangdong of China, comparable to the findings in the UK (8.28%, 2006–2010) (22) and Bangladesh (8.4%, 2014) (23) but lower than the United States (14.4%, 2018) (9). The higher US prevalence may reflect its aging population structure and greater chronic disease risk factor prevalence (24). We further identified a significant upward trend of CMM prevalence in Guangdong during the study period (4.31 to 8.75% during 2007–2018), consistent with US patterns (9.4 to 14.4% during 1999–2018) (9). However, the average annual growth rate (0.40%) in Guangdong exceeded that in the United States (0.26%), potentially attributable to accelerated population aging and rapid lifestyle changes driven by economic development in Guangdong Province. For example, census data indicated Guangdong’s population aged ≥65 years increased from 6.79 to 9.96% between 2010 and 2023. Additionally, rapid economic development coincided with increased unhealthy lifestyles—including reduced physical activity, greater sedentary time, and poorer dietary patterns (25). This finding indicates that CMM may be a critical public health challenge in Guangdong Province amid dual challenges of accelerated population aging and lifestyle changes in the future.

We further found that individuals with CMM faced elevated mortality risk, with higher risk for those with more CMDs, consistent with prior studies (2, 26, 27). For instance, the Emerging Risk Factors Collaboration reported HRs of approximately 2, 4, and 7 for individuals with 1, 2, and 3 CMDs, respectively (2). The Jackson Heart Study found the combination of T2D, stroke, and CHD associated with the highest all-cause mortality (HR = 3.68) (27). Similarly, among older US adults, the combination of myocardial infarction, stroke, and T2D yielded an HR of 4.74 (28). These findings suggest CMM may cause cumulative damage to multiple organ systems, leading to functional failure and increased mortality (29). Furthermore, managing CMM presents greater clinical challenges due to potential polypharmacy interactions, substantially complicating treatment regimens and contributing to adverse outcomes. This finding underscores the critical need for personalized CMM care strategies to mitigate therapeutic complexities and improve long-term prognosis.

We further observed that mortality risk associated with CMM was higher in patients aged <65 years than in those aged ≥65 years, indicating greater risk for early-onset versus late-onset CMM. This disparity may reflect pathophysiological differences across age groups. For instance, studies show atherosclerotic plaques in younger patients typically feature lipid-rich cores, thin fibrous caps, and active inflammation, predisposing to acute thrombosis (e.g., acute coronary syndrome). In contrast, plaques in older adults often demonstrate calcification, fibrosis, and structural stability, with lower acute risk but increased chronic ischemia due to progressive stenosis (30). We also found urban residents had higher CMM-related mortality risk than rural residents, and the underlying reasons were unclear, which is deserved to conduct deep research in the future.

While most previous studies have examined CMM–mortality associations, cross-sectional design limitations have left a critical knowledge gap regarding CMM trajectory–mortality relationships (26, 31). Our cohort study revealed that the “healthy-C-M” progression trajectory carried the highest mortality risk among all trajectories. Although few studies have explored this, several mechanisms may explain it. First, cardiovascular diseases cause irreversible organ damage even in early stages. Subsequent metabolic disorders (e.g., diabetes) can trigger excessive glucose influx into mitochondria, inducing superoxide production and exacerbating oxidative stress (32). Increased advanced glycation end products (AGEs) elevate cardiovascular tissue stiffness (33), accelerating functional decline. Previous evidence indicates diabetes increases coronary heart disease mortality risk by 59% (34). Second, cardiovascular medications (beta-blockers and diuretics) may worsen insulin resistance or mask hypoglycemia symptoms (35). Additionally, heart failure patients’ limited mobility increases susceptibility to metabolic disorders (36), accelerating CMM progression. In contrast, early metabolic disorders rarely cause immediate organ damage, and early prevention can slow progression and reduce mortality. For example, controlled hypertension (<140/90 mmHg) shows comparable all-cause mortality to normotensive individuals (37). These findings provide new insights into CMM progression, suggesting primary focus should be on the “healthy-C-M” trajectory to improve patient outcomes in Guangdong Province. Despite China’s 2009 integration of chronic disease management into the National Essential Public Health Services (NEPHS), current protocols primarily focus on single-disease paradigm, and a paradigm shift toward integrated multimorbidity care is imperative. Stratified analyses indicated that females with “healthy-M-C” trajectory had higher mortality risk, which could potentially be explained by the waning estrogen’s protective effect for cardiovascular health (38). Cardiovascular symptoms in women often present atypically (such as fatigue, shoulder/back pain, and nausea) rather than classic chest pain, leading to misdiagnosis or delayed treatment (39).

Although current evidence does not define distinct pharmacological protocols for individual CMM trajectories, core cardiometabolic interventions (e.g., blood pressure control, glycemic management, lipid lowering, and lifestyle optimization) are recommended regardless of the sequence of disease onset. Nevertheless, the markedly divergent mortality risks across trajectories imply that the timing and intensity of preventive care could usefully be informed by trajectory patterns. For instance, patients who first present with cardiovascular disease may benefit from prioritized metabolic monitoring and early initiation of glucose-lowering strategies to delay or prevent progression to CMM. Trajectory-based risk stratification thus offers a practical framework for allocation of clinical vigilance, even if the pharmacological components overlap. Prospective studies are warranted to test whether trajectory-tailored interventions can improve outcomes compared with uniform multimorbidity management.

The study has several strengths. First, based on data from Guangdong Chronic Disease and Risk Factor Surveillance during 2007–2018, this study established a cohort named as GDACRC. The broad age range and diverse sociodemographic characteristics of participants enhance the representativeness of our study. Second, we described the temporal trend of age-sex standardized CMM prevalence in China based on five cross-sectional survey waves. Third, the large number of deaths recorded during more than 15 years of follow-up allowed us to investigate the associations between CMM trajectories and mortality in China. However, several limitations should be acknowledged in our study. First, our analysis only involved participants from a province, potentially limiting the generalization of our results. Second, individual behavior and lifestyle may change over time, and residual confounding elements may persist despite our efforts to control them. Third, in the baseline survey, the occurrence of T2D, hypertension, CHD, and stroke was based on self-report, which may lead to information bias. Fourth, mortality was ascertained from the provincial cause-of-death registry, which has very low underreporting owing to mandatory death certification. However, a small number of out-of-province deaths may have been missed, although our cohort of permanent residents limits this concern. Incident CMD was identified from hospitalization records and may miss the hospitalizations outside Guangdong, potentially leading to underestimation of CMD incidence. Fifth, the CMM definition was limited to T2D, CHD, stroke, and hypertension. Clinically relevant conditions, such as chronic kidney disease, could not be included because the necessary biomarkers were not collected at baseline. Consequently, the prevalence and trajectory patterns reported here may underestimate the broader spectrum of cardiometabolic multimorbidity. Sixth, the trajectory analysis was restricted to participants free of any CMD at baseline. This ensured a homogeneous starting point but excluded the progression from a single pre-existing CMD to CMM, limiting the generalizability of the identified trajectories to populations already living with one cardiometabolic disease.

## Conclusion

CMM represents a major public health challenge in Guangdong Province, China, with higher mortality risk on patients with CMM and “healthy-C-M” trajectory. Our findings could inform developing more targeted strategies on prevention and clinical management of CMM in China.

## Data Availability

The original contributions presented in the study are included in the article/Supplementary material, further inquiries can be directed to the corresponding authors.
