# Supplementary material for: The trajectories of cardiometabolic multimorbidity associate with mortality risk: a study based on Guangdong adult chronic diseases and risk factors cohort
Source: Front Public Health. 2026 Jul 9;14:1833156. doi: 10.3389/fpubh.2026.1833156 (PMC13391277; doi:10.3389/fpubh.2026.1833156)
Supplement: Supplementary file 1 [file Data_Sheet_1.DOCX]

**Supplemental Materials**

**The trajectories of Cardiometabolic Multimorbidity (CMM) associate with Mortality Risk: a study based on Guangdong adult chronic diseases and risk factors cohort**

[Figure S1 Conceptual Framework of Baseline-hospitalization-death data 5](#_Toc229951932)

[Table S1 Demographic Characteristics (Age and Sex) in Different Survey Rounds 6](#_Toc229951933)

[Figure S2 The temporal trend of age-sex standardized CMD and CMM prevalence from 2007 to 2018 in Guangdong province. HBP: Hypertension; T2D: Type 2 Diabetes; CHD: Coronary Heart Disease. A: Tends in prevalence of single CMD (Cardiometabolic Disease) from 2007 to 2018; B: Tends in prevalence of CMM (Cardiometabolic Multimorbidity) with 2 CMDs from 2007 to 2018; C: Tends in prevalence of CMM with 3 CMDs from 2007 to 2018. 7](#_Toc229951934)

[Table S2 The temporal trend of standardized CMD and CMM prevalence from 2007 to 2018 in Guangdong province 8](#_Toc229951935)

[Table S3 The temporal trend of standardized CMD and CMM prevalence from 2007 to 2018 in Guangdong province in males and females 9](#_Toc229951936)

[Table S4 The temporal trend of standardized CMD and CMM prevalence from 2007 to 2018 in Guangdong province in participants aged <65 and aged ≥65 11](#_Toc229951937)

[Table S5 The temporal trend of standardized CMD and CMM prevalence from 2007 to 2018 in Guangdong province in rural and urban residents 13](#_Toc229951938)

[Table S6 The association between CMM status and mortality 15](#_Toc229951939)

[Table S7 The associations between the number of CMD and mortality 17](#_Toc229951940)

[Table S8 The associations between different multimorbidity development trajectories and mortality 19](#_Toc229951941)

[Table S9 Multiplicity-adjusted P values for interaction tests between CMM status/trajectory groups and stratification variables on all-cause mortality 21](#_Toc229951942)

[Figure S3 Associations between CMM status and all‑cause mortality across subgroups (follow‑up ≥12 years). 22](#_Toc229951943)

[Figure S4 Associations between CMM trajectories and all‑cause mortality across subgroups (follow‑up ≥12 years). 23](#_Toc229951944)

[Table S10 The temporal trend of standardized CMD and CMM prevalence from 2007 to 2018 in Guangdong province 24](#_Toc229951945)

[Table S11 Sensitivity analysis restricting to different age ranges: associations of CMM status and trajectories with all-cause mortality among adults aged over 18, 35, and 40 25](#_Toc229951946)

[Table S12 E-values for the associations of CMD status and trajectory with all‑cause mortality, overall and by subgroups 25](#_Toc229951947)

[Table S13 Schoenfeld residual test for proportional hazards assumption of full follow‑up period 26](#_Toc229951948)

[Table S14 Schoenfeld residual test for proportional hazards assumption after restricting follow‑up to 0–12year window 26](#_Toc229951949)

[Table S15 Segmented Cox regression analysis: associations of CMD status with all-cause mortality stratified by 4-year intervals 27](#_Toc229951950)

[Table S16 Segmented Cox regression analysis: associations of CMM trajectories with all-cause mortality stratified by 4-year intervals 27](#_Toc229951951)


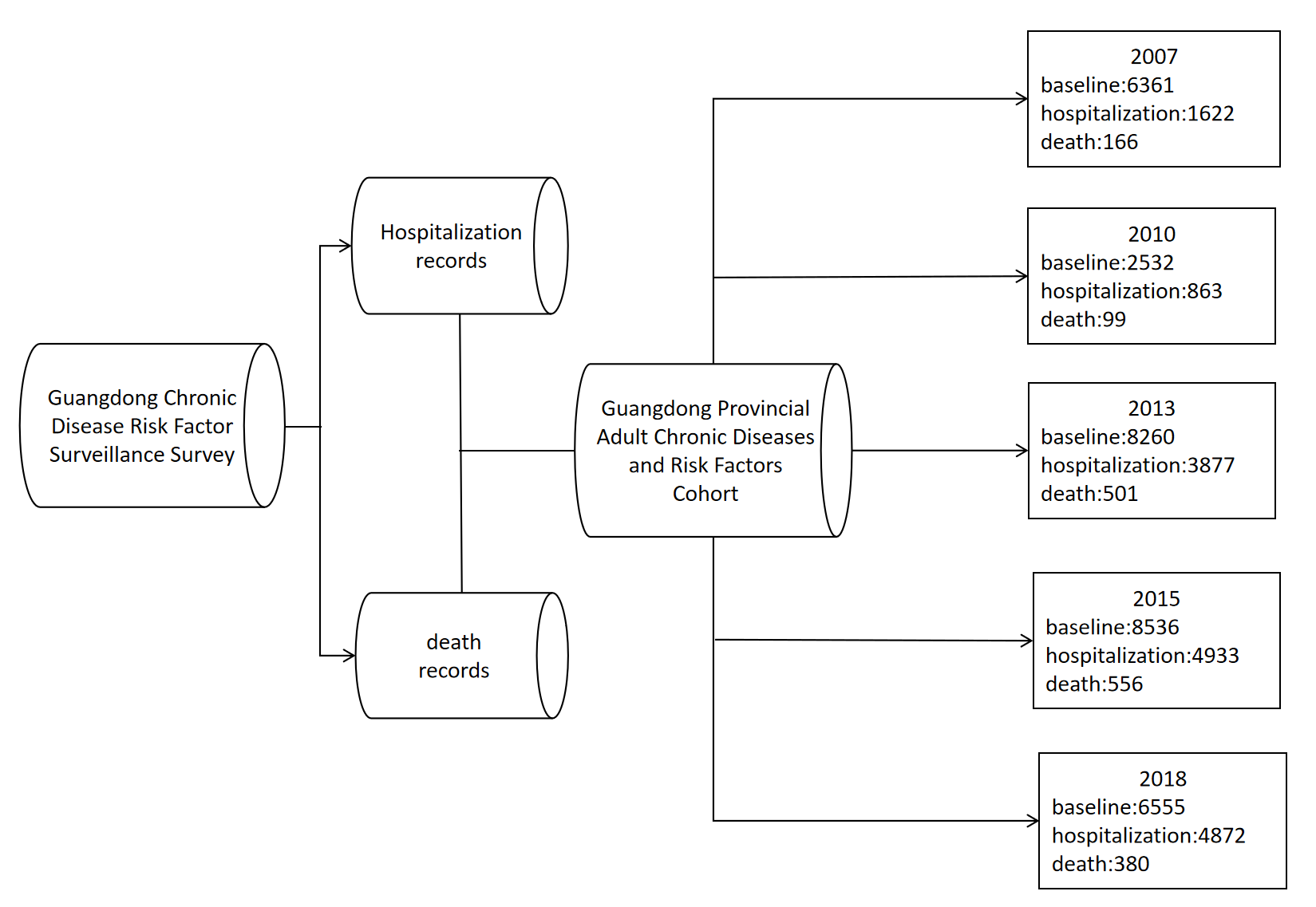


# Figure S1 **Conceptual Framework of Baseline-hospitalization-death data**

Table S1 Schoenfeld residual test for proportional hazards assumption of full follow‑up period

| Model | χ² | P value |
| --- | --- | --- |
| CMM status | 88.97 | <0.001 |
| CMM trajectory | 38.02 | 0.002 |

The proportional hazards assumption was tested using scaled Schoenfeld residuals. P < 0.05 suggests potential non‑proportionality.

# Table S2 Segmented Cox regression analysis: associations of CMD status with all-cause mortality stratified by 4-year intervals

| CMM status | HR(95%CI) | | | |
| --- | --- | --- | --- | --- |
|  | 0-4 years | 4-8 years | 8-12 years | >12 years |
| Without CMD | 1.00 (REF) | | | |
| Single CMD | 1.62(1.25, 2.09) | 1.51(1.24,1.87) | 1.27(0.95, 1.71) | 1.43(0.90, 2.27) |
| CMM | 2.53(1.95,3.27) | 2.84(2.32, 3.49) | 2.64(1.98, 3.52) | 3.39(2.10, 5.35) |

# Table S3 Segmented Cox regression analysis: associations of CMM trajectories with all-cause mortality stratified by 4-year intervals

| CMM trajectory | HR(95%CI) | | | |
| --- | --- | --- | --- | --- |
|  | 0-4 years | 4-8 years | 8-12 years | >12 years |
| H→M | 1.00 (REF) | | | |
| H→C | 1.97(0.98, 3.73) | 1.93(1.12,3.36) | 1.83(1.04, 3.35) | 2.42(0.94,6.31) |
| H→M→CMM | 4.90(0.10,2.20) | 1.69(1.07,2.39) | 2.48(1.14,5.40) | 6.25(2.45,17.34) |
| H→C→CMM | 2.00(0.82,4.84) | 2.83(1.50,5.50) | 4.12(1.93,8.80) | 6.03(2.07,23.06) |

Table S4 Schoenfeld residual test for proportional hazards assumption after restricting follow‑up to 0–12year window

| Model | χ² | P value |
| --- | --- | --- |
| CMM status | 20.39 | 0.118 |
| CMM trajectory | 21.55 | 0.120 |

The proportional hazards assumption was tested using scaled Schoenfeld residuals. P < 0.05 suggests potential non‑proportionality.

# Table S5 Demographic Characteristics (Age and Sex) in Different Survey Rounds

| Variable | 2007 | 2010 | 2013 | 2015 | 2018 |
| --- | --- | --- | --- | --- | --- |
| sex |  |  |  |  |  |
| male | 3023(47.53%) | 1132(44.71%) | 3528(42.71%) | 3793(44.44%) | 3713(43.39%) |
| female | 3338(52.47%) | 1400(55.29%) | 4732(57.29%) | 4743(55.56%) | 4832(56.61%) |
| age | 43.69±13.38 | 49.26±16.15 | 53.28±14.32 | 51.97±14.97 | 54.23±13.85 |
| Total | 6361 | 2532 | 8260 | 8536 | 8555 |


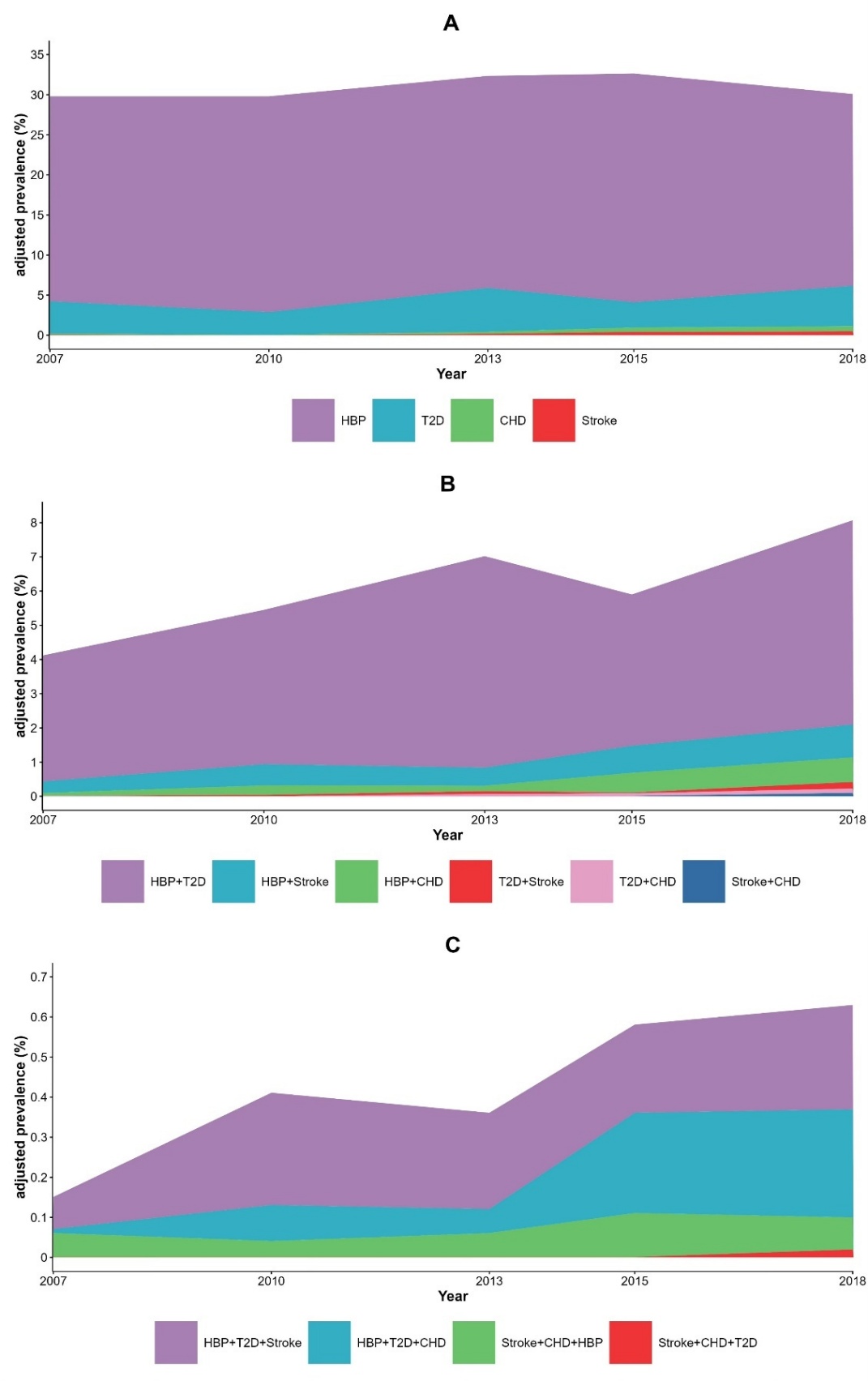


# Figure S2 The temporal trend of age-sex standardized CMD and CMM prevalence from 2007 to 2018 in Guangdong province. HBP: Hypertension; T2D: Type 2 Diabetes; CHD: Coronary Heart Disease. A: Tends in prevalence of single CMD (Cardiometabolic Disease) from 2007 to 2018; B: Tends in prevalence of CMM (Cardiometabolic Multimorbidity) with 2 CMDs from 2007 to 2018; C: Tends in prevalence of CMM with 3 CMDs from 2007 to 2018.

# Table S6 The temporal trend of standardized CMD and CMM prevalence from 2007 to 2018 in Guangdong province

| CMD/CMM | Adjusted prevalence (%) | | | | |
| --- | --- | --- | --- | --- | --- |
|  | 2007 | 2010 | 2013 | 2015 | 2018 |
| HBP | 25.56(24.49,26.63) | 26.91(25.18,28.64) | 26.45(25.50,27.40) | 28.53(27.57,29.49) | 23.92(23.02,24.82) |
| T2D | 4.04(3.56,4.52) | 2.84(2.19,3.49) | 5.46(4.97,5.95) | 3.15(2.78,3.52) | 5.04(4.58,5.50) |
| CHD | 0.12(0.03,0.21) | 0.04(-0.04,0.12) | 0.27(0.16,0.38) | 0.56(0.40,0.72) | 0.66(0.49,0.83) |
| Stroke | 0.08(0.01,0.15) | 0.00(0.00,0.00) | 0.17(0.08,0.26) | 0.41(0.27,0.55) | 0.47(0.33,0.61) |
| Single CMD | 29.80(28.67,30.93) | 29.79(28.01,31.57) | 32.35(32.34,32.65) | 32.65(31.66,33.64) | 30.10(29.13,31.07) |
| HBP+T2D | 3.68(3.22,4.14) | 4.51(3.70,5.32) | 6.18(5.66,6.70) | 4.42(3.98,4.86) | 5.97(5.47,6.47) |
| HBP+Stroke | 0.34(0.20,0.48) | 0.62(0.31,0.93) | 0.53(0.37,0.69) | 0.79(0.60,0.98) | 0.96(0.75,1.17) |
| HBP+CHD | 0.08(0.01,0.15) | 0.28(0.07,0.49) | 0.16(0.07,0.25) | 0.58(0.42,0.74) | 0.72(0.54,0.90) |
| T2D+Stroke | 0.00(0.00,0.00) | 0.04(-0.04,0.12) | 0.08(0.02,0.14) | 0.02(-0.01,0.03) | 0.19(0.10,0.28) |
| T2D+CHD | 0.01(-0.01,0.03) | 0.00(0.00,0.00) | 0.06(0.01,0.11) | 0.08(0.02,0.14) | 0.13(0.05,0.21) |
| Stroke+CHD | 0.01(-0.01,0.03) | 0.00(0.00,0.00) | 0.01(-0.01,0.03) | 0.01(-0.01,0.03) | 0.10(0.03,0.17) |
| CMM with 2 diseases | 4.12(3.63,4.61) | 5.45(4.57,6.35) | 7.02(6.46,7.56) | 5.90(5.40,6.40) | 8.08(7.50,8.66) |
| HBP+T2D+Stroke | 0.08(0.01,0.15) | 0.28(0.07,0.49) | 0.24(0.13,0.35) | 0.22(0.12,0.32) | 0.26(0.15,0.37) |
| HBP+T2D+CHD | 0.01(-0.01,0.03) | 0.09(-0.03,0.21) | 0.06(0.01,0.11) | 0.25(0.14,0.36) | 0.27(0.16,0.38) |
| Stroke+CHD+HBP | 0.06(0.00,0.12) | 0.04(-0.04,0.12) | 0.06(0.01,0.11) | 0.11(0.04,0.18) | 0.08(0.02,0.14) |
| Stroke+CHD+T2D | 0.00(0.00,0.00) | 0.00(0.00,0.00) | 0.00(0.00,0.00) | 0.00(0.00,0.00) | 0.02(-0.01,0.05) |
| CMM with 3 diseases | 0.15(0.05,0.25) | 0.40(0.15,0.65) | 0.35(0.22,0.48) | 0.59(0.43,0.75) | 0.63(0.46,0.80) |
| CMM≥2 diseases | 4.31(3.81,4.81) | 5.86(4.94,6.78) | 7.36(6.80,7.92) | 6.50(5.98,7.02) | 8.75(8.15,9.35) |

# Table S7 The temporal trend of standardized CMD and CMM prevalence from 2007 to 2018 in Guangdong province in males and females

|  | male | | | | |  | female | | | | |
| --- | --- | --- | --- | --- | --- | --- | --- | --- | --- | --- | --- |
| CMD/CMM | Adjusted prevalence (%) | | | | |  | Adjusted prevalence (%) | | | | |
|  | 2007 | 2010 | 2013 | 2015 | 2018 |  | 2007 | 2010 | 2013 | 2015 | 2018 |
| HBP | 21.63  (20.16,23.09) | 28.88  (26.23,31.52) | 27.89  (26.41,29.37) | 31.29  (29.82,32.77) | 25.75  (24.35,27.16) |  | 22.99  (21.56,24.42) | 24.79  (22.52,27.05) | 26.16  (24.91,27.41) | 27.07  (25.81,28.34) | 24.33  (23.13,25.54) |
| T2D | 4.08  (3.37,4.79) | 3.37  (2.31,4.42) | 5.56  (4.80,6.31) | 3.22  (2.65,3.78) | 5.66  (4.91,6.40) |  | 3.88  (3.22,4.53) | 2.44  (1.63,3.24) | 5.33  (4.69,5.97) | 3.06  (2.57,3.55) | 4.57  (3.98,5.15) |
| CHD | 0.17  (0.02,0.31) | 0.00  (0.00,0.00) | 0.26  (0.09,0.42) | 0.71  (0.44,0.98) | 0.84  (0.54,1.13) |  | 0.06  (-0.02,0.14) | 0.07  (-0.07,0.21) | 0.30  (0.14,0.45) | 0.44  (0.25,0.63) | 0.62  (0.40,0.84) |
| Stroke | 0.17  (0.02,0.31) | 0.00  (0.00,0.00) | 0.28  (0.11,0.46) | 0.47  (0.26,0.69) | 0.67  (0.41,0.94) |  | 0.03  (-0.03,0.09) | 0.00  (0.00,0.00) | 0.08  (0,0.17) | 0.36  (0.19,0.53) | 0.39  (0.22,0.57) |
| Single CMD | 26.04  (24.47,27.60) | 32.24  (29.51,34.97) | 33.99  (32.42,35.55) | 35.7  (24.17,37.22) | 32.92  (31.41,34.43) |  | 26.95  (25.45,28.46) | 27.29  (24.96,29.63) | 31.87  (30.54,33.20) | 30.93  (29.61,32.25) | 29.91  (28.62,31.20) |
| HBP+T2D | 2.49  (1.93,3.04) | 4.87  (3.62,6.13) | 5.81  (5.04,6.58) | 4.43  (3.77,5.08) | 6.28  (5.50,7.06) |  | 2.94  (2.37,3.52) | 4.08  (3.04,5.12) | 6.74  (6.03,7.46) | 4.55  (3.96,5.15) | 6.42  (5.73,7.11) |
| HBP+Stroke | 0.33  (0.13,0.54) | 0.97  (0.40,1.55) | 0.96  (0.64,1.29) | 1.13  (0.8,1.47) | 1.64  (1.23,2.05) |  | 0.15  (0.02,0.28) | 0.29  (0.01,0.57) | 0.21  (0.08,0.34) | 0.55  (0.34,0.76) | 0.64  (0.42,0.87) |
| HBP+CHD | 0.10  (-0.01,0.21) | 0.27  (-0.03,0.57) | 0.20  (0.05,0.35) | 0.55  (0.32,0.79) | 0.97  (0.65,1.29) |  | 0.03  (-0.03,0.09) | 0.29  (0.01,0.57) | 0.13  (0.03,0.23) | 0.63  (0.41,0.86) | 0.68  (0.45,0.91) |
| T2D+Stroke | 0.00  (0.00,0.00) | 0.00  (0.00,0.00) | 0.11  (0.00,0.22) | 0.05  (-0.02,0.13) | 0.19  (0.05,0.33) |  | 0.00  (0.00,0.00) | 0.07  (-0.07,0.21) | 0.06  (-0.01,0.14) | 0.00  (0.00,0.00) | 0.21  (0.08,0.33) |
| T2D+CHD | 0.03  (-0.03,0.10) | 0.00  (0.00,0.00) | 0.09  (-0.01,0.18) | 0.08  (-0.01,0.17) | 0.19  (0.05,0.33) |  | 0.00  (0.00,0.00) | 0.00  (0.00,0.00) | 0.04  (-0.02,0.10) | 0.08  (0,0.17) | 0.10  (0.01,0.19) |
| Stroke+CHD | 0.03  (-0.03,0.10) | 0.00  (0.00,0.00) | 0.00  (0.00,0.00) | 0.03  (-0.03,0.08) | 0.22  (0.07,0.36) |  | 0.00  (0.00,0.00) | 0.00  (0.00,0.00) | 0.02  (-0.02,0.06) | 0.00  (0.00,0.00) | 0.02  (-0.02,0.06) |
| CMM with 2 diseases | 2.99  (2.38,3.59) | 6.11  (4.71,7.51) | 7.17  (6.32,8.02) | 6.27  (5.50,7.05) | 9.48  (8.54,10.43) |  | 3.13  (2.53,3.72) | 4.73  (3.61,5.84) | 7.21  (6.47,7.94) | 5.82  (5.15,6.49) | 8.08  (7.31,8.84) |
| HBP+T2D+Stroke | 0.13  (0,0.26) | 0.27  (-0.03,0.57) | 0.31  (0.13,0.50) | 0.37  (0.18,0.56) | 0.48  (0.26,0.71) |  | 0.00  (0.00,0.00) | 0.29  (0.01,0.57) | 0.19  (0.07,0.31) | 0.13  (0.03,0.23) | 0.12  (0.02,0.22) |
| HBP+T2D+CHD | 0.03  (-0.03,0.10) | 0.09  (-0.08,0.26) | 0.09  (-0.01,0.18) | 0.29  (0.12,0.46) | 0.13  (0.02,0.25) |  | 0.00  (0.00,0.00) | 0.07  (-0.07,0.21) | 0.04  (-0.02,0.10) | 0.23  (0.1,0.37) | 0.43  (0.25,0.62) |
| Stroke+CHD+HBP | 0.00  (0.00,0.00) | 0.09  (-0.08,0.26) | 0.09  (-0.01,0.18) | 0.08  (-0.01,0.17) | 0.13  (0.02,0.25) |  | 0.06  (-0.02,0.14) | 0.00  (0.00,0.00) | 0.04  (-0.02,0.10) | 0.15  (0.04,0.26) | 0.08  (0.00,0.16) |
| Stroke+CHD+T2D | 0.00  (0.00,0.00) | 0.00  (0.00,0.00) | 0.00  (0.00,0.00) | 0.00  (0.00,0.00) | 0.03  (-0.03,0.08) |  | 0.00  (0.00,0.00) | 0.00  (0.00,0.00) | 0.00  (0.00,0.00) | 0.00  (0.00,0.00) | 0.02  (-0.02,0.06) |
| CMM with 3 diseases | 0.17  (0.02,0.31) | 0.44  (0.06,0.83) | 0.48  (0.25,0.71) | 0.74  (0.47,1.01) | 0.78  (0.50,1.06) |  | 0.06  (-0.02,0.14) | 0.36  (0.04,0.67) | 0.27  (0.13,0.42) | 0.51  (0.3,0.71) | 0.66  (0.43,0.89) |
| CMM≥2 diseases | 3.01  (2.41,3.60) | 7.24  (5.65,8.83) | 6.49  (5.74,7.23) | 7.33  (6.48,8.17) | 10.54  (9.54,11.54) |  | 3.22  (2.62,3.81) | 5.09  (3.93,6.24) | 7.48  (6.73,8.23) | 6.33  (5.63,7.02) | 8.76  (7.96,9.55) |

# Table S8 The temporal trend of standardized CMD and CMM prevalence from 2007 to 2018 in Guangdong province in participants aged <65 and aged ≥65

|  | aged <65 | | | | |  | aged ≥65 | | | | |
| --- | --- | --- | --- | --- | --- | --- | --- | --- | --- | --- | --- |
| CMD/CMM | Adjusted prevalence (%) | | | | |  | Adjusted prevalence (%) | | | | |
|  | 2007 | 2010 | 2013 | 2015 | 2018 |  | 2007 | 2010 | 2013 | 2015 | 2018 |
| HBP | 20.73  (19.7,21.77) | 22.32  (20.53,24.11) | 22.35  (21.34,23.36) | 23.44  (22.44,24.45) | 20.73  (19.75,21.72) |  | 45.81  (40.97,50.66) | 46.64  (42.01,51.27) | 44.68  (42.31,47.06) | 50.97  (48.6,53.34) | 38.32  (36.22,40.43) |
| T2D | 3.96  (3.46,4.45) | 2.84  (2.12,3.55) | 5.75  (5.18,6.31) | 3.34  (2.91,3.76) | 5.09  (4.56,5.63) |  | 4.19  (2.24,6.14) | 2.91  (1.35,4.48) | 4.16  (3.21,5.11) | 2.28  (1.58,2.99) | 4.88  (3.94,5.81) |
| CHD | 0.10  (0.02,0.18) | 0.00  (0.00,0.00) | 0.18  (0.08,0.29) | 0.51  (0.34,0.68) | 0.54  (0.36,0.72) |  | 0.25  (-0.24,0.73) | 0.22  (-0.21,0.66) | 0.65  (0.27,1.04) | 0.76  (0.35,1.17) | 1.27  (0.78,1.75) |
| Stroke | 0.10  (0.02,0.18) | 0.00  (0.00,0.00) | 0.14  (0.05,0.23) | 0.38  (0.23,0.53) | 0.35  (0.21,0.5) |  | 0.00  (0.00,0.00) | 0.00  (0.00,0.00) | 0.30  (0.04,0.56) | 0.53  (0.18,0.87) | 1.02  (0.59,1.46) |
| Single CMD | 24.89  (23.79,25.99) | 25.16  (23.29,27.02) | 28.42  (27.33,29.51) | 27.68  (26.61,28.74) | 26.71  (25.64,27.79) |  | 50.25  (45.38,55.11) | 49.78  (45.14,54.42) | 49.79  (47.4,52.18) | 54.54  (52.18,56.90) | 45.49  (43.33,47.65) |
| HBP+T2D | 2.21  (1.83,2.58) | 3.32  (2.55,4.09) | 5.03  (4.5,5.56) | 3.43  (3,3.86) | 4.97  (4.44,5.50) |  | 10.34  (7.38,13.31) | 9.64  (6.9,12.38) | 11.47  (9.95,12.99) | 8.79  (7.44,10.13) | 10.78  (9.43,12.12) |
| HBP+Stroke | 0.19  (0.08,0.29) | 0.34  (0.09,0.59) | 0.35  (0.21,0.49) | 0.50  (0.33,0.66) | 0.60  (0.41,0.79) |  | 0.99  (0.02,1.95) | 1.79  (0.56,3.03) | 1.25  (0.72,1.78) | 2.05  (1.38,2.72) | 2.58  (1.90,3.27) |
| HBP+CHD | 0.05  (-0.01,0.11) | 0.19  (0.00,0.38) | 0.12  (0.04,0.21) | 0.34  (0.2,0.47) | 0.46  (0.3,0.63) |  | 0.25  (-0.24,0.73) | 0.67  (-0.09,1.43) | 0.30  (0.04,0.56) | 1.64  (1.04,2.24) | 1.90  (1.31,2.49) |
| T2D+Stroke | 0.00  (0.00,0.00) | 0.00  (0.00,0.00) | 0.05  (-0.01,0.10) | 0.03  (-0.01,0.07) | 0.17  (0.07,0.27) |  | 0.00  (0.00,0.00) | 0.22  (-0.21,0.66) | 0.24  (0.01,0.47) | 0.00  (0.00,0.00) | 0.29  (0.06,0.53) |
| T2D+CHD | 0.02  (-0.02,0.05) | 0.00  (0.00,0.00) | 0.03  (-0.01,0.07) | 0.04  (-0.01,0.09) | 0.12  (0.04,0.21) |  | 0.00  (0.00,0.00) | 0.00  (0.00,0.00) | 0.18  (-0.02,0.38) | 0.23  (0,0.46) | 0.20  (0.00,0.39) |
| Stroke+CHD | 0.02  (-0.02,0.05) | 0.00  (0.00,0.00) | 0.02  (-0.01,0.05) | 0.00  (0.00,0.00) | 0.06  (0.00,0.12) |  | 0.00  (0.00,0.00) | 0.00  (0.00,0.00) | 0.00  (0.00,0.00) | 0.06  (-0.06,0.17) | 0.24  (0.03,0.46) |
| CMM with 2 diseases | 2.48  (2.08,2.87) | 3.85  (3.02,4.67) | 5.60  (5.04,6.15) | 4.33  (3.85,4.82) | 6.38  (5.79,6.98) |  | 11.58  (8.46,14.69) | 12.33  (9.28,15.38) | 13.43  (11.8,15.06) | 12.77  (11.19,14.35) | 15.99  (14.41,17.58) |
| HBP+T2D+Stroke | 0.05  (-0.01,0.11) | 0.19  (0.00,0.38) | 0.15  (0.06,0.25) | 0.13  (0.05,0.22) | 0.2  (0.09,0.31) |  | 0.25  (-0.24,0.73) | 0.67  (-0.09,1.43) | 0.59  (0.23,0.96) | 0.64  (0.26,1.02) | 0.54  (0.22,0.85) |
| HBP+T2D+CHD | 0.02  (-0.02,0.05) | 0.00  (0.00,0.00) | 0.02  (-0.01,0.05) | 0.19  (0.09,0.29) | 0.15  (0.06,0.25) |  | 0.00  (0.00,0.00) | 0.45  (-0.17,1.07) | 0.24  (0.01,0.47) | 0.53  (0.18,0.87) | 0.78  (0.40,1.16) |
| Stroke+CHD+HBP | 0.02  (-0.02,0.05) | 0.05  (-0.05,0.14) | 0.02  (-0.01,0.05) | 0.06  (0.00,0.12) | 0.02  (-0.01,0.05) |  | 0.25  (-0.24,0.73) | 0.00  (0.00,0.00) | 0.24  (0.01,0.47) | 0.35  (0.07,0.63) | 0.39  (0.12,0.66) |
| Stroke+CHD+T2D | 0.00  (0.00,0.00) | 0.00  (0.00,0.00) | 0.00  (0.00,0.00) | 0.00  (0.00,0.00) | 0.00  (0.00,0.00) |  | 0.00  (0.00,0.00) | 0.00  (0.00,0.00) | 0.00  (0.00,0.00) | 0.00  (0.00,0.00) | 0.10  (-0.04,0.23) |
| CMM with 3 diseases | 0.08  (0.01,0.16) | 0.24  (0.03,0.45) | 0.18  (0.08,0.29) | 0.38  (0.23,0.53) | 0.37  (0.22,0.52) |  | 0.49  (-0.19,1.17) | 1.12  (0.14,2.10) | 1.07  (0.58,1.56) | 1.52  (0.94,2.10) | 1.80  (1.23,2.38) |
| CMM≥2 diseases | 2.56  (2.16,2.96) | 4.09  (3.24,4.94) | 5.78  (5.21,6.34) | 4.73  (4.23,5.23) | 6.77  (6.16,7.38) |  | 12.32  (9.12,15.51) | 13.45  (10.29,16.62) | 14.5  (12.82,16.18) | 14.29  (12.63,15.95) | 17.94  (16.28,19.60) |

# Table S9 The temporal trend of standardized CMD and CMM prevalence from 2007 to 2018 in Guangdong province in rural and urban residents

|  | rural | | | | |  | urban | | | | |
| --- | --- | --- | --- | --- | --- | --- | --- | --- | --- | --- | --- |
| CMD/CMM | Adjusted prevalence  (%) | | | | |  | Adjusted prevalence  (%) | | | | |
|  | 2007 | 2010 | 2013 | 2015 | 2018 |  | 2007 | 2010 | 2013 | 2015 | 2018 |
| HBP | 21.75  (20.31,23.18) | 17.84  (15.91,19.78) | 26.63  (25.28,27.98) | 25.15  (23.93,26.36) | 19.51  (18.4,20.62) |  | 22.78  (21.33,24.24) | 26.90  (24.65,29.14) | 27.61  (26.24,28.97) | 25.37  (24.15,26.59) | 23.95  (22.76,25.14) |
| T2D | 4.15  (3.46,4.84) | 2.06  (1.34,2.78) | 5.49  (4.79,6.19) | 2.23  (1.81,2.64) | 4.09  (3.54,4.65) |  | 3.77  (3.11,4.43) | 2.73  (1.91,3.55) | 5.44  (4.75,6.14) | 3.23  (2.73,3.73) | 4.68  (4.09,5.28) |
| CHD | 0.09  (-0.01,0.20) | 0.07  (-0.06,0.20) | 0.34  (0.16,0.52) | 0.29  (0.14,0.44) | 0.49  (0.29,0.68) |  | 0.13  (0.00,0.25) | 0.00  (0.00,0.00) | 0.22  (0.08,0.36) | 0.70  (0.46,0.93) | 0.75  (0.51,10) |
| Stroke | 0.09  (-0.01,0.20) | 0.00  (0.00,0.00) | 0.17  (0.04,0.3) | 0.35  (0.18,0.51) | 0.63  (0.41,0.85) |  | 0.09  (-0.01,0.20) | 0.00  (0.00,0.00) | 0.17  (0.04,0.30) | 0.37  (0.20,0.54) | 0.26  (0.12,0.41) |
| Single CMD | 26.26  (24.72,27.79) | 29.35  (26.56,32.15) | 32.12  (30.7,33.53) | 37.59  (36.01,39.16) | 33.32  (31.79,34.85) |  | 26.78  (25.24,28.31) | 29.63  (27.32,31.94) | 33.44  (31.99,34.88) | 29.67  (28.39,30.95) | 29.65  (28.38,30.93) |
| HBP+T2D | 2.36  (1.83,2.88) | 2.20  (1.46,2.94) | 6.49  (5.74,7.25) | 3.76  (3.23,4.30) | 5.32  (4.69,5.94) |  | 3.08  (2.48,3.68) | 5.26  (4.13,6.39) | 6.30  (5.55,7.04) | 4.09  (3.53,4.64) | 5.76  (5.11,6.42) |
| HBP+Stroke | 0.16  (0.02,0.29) | 0.47  (0.12,0.81) | 0.51  (0.29,0.73) | 0.63  (0.41,0.86) | 0.88  (0.62,1.14) |  | 0.31  (0.12,0.51) | 0.53  (0.16,0.90) | 0.56  (0.33,0.79) | 0.78  (0.53,1.02) | 1.00  (0.72,1.28) |
| HBP+CHD | 0.09  (-0.01,0.20) | 0.20  (-0.03,0.43) | 0.17  (0.04,0.30) | 0.27  (0.12,0.41) | 0.59  (0.38,0.8) |  | 0.03  (-0.03,0.09) | 0.27  (0.01,0.53) | 0.15  (0.03,0.26) | 0.78  (0.53,1.02) | 0.81  (0.56,1.07) |
| T2D+Stroke | 0.00  (0.00,0.00) | 0.07  (-0.06,0.20) | 0.10  (0.00,0.19) | 0.00  (0.00,0.00) | 0.16  (0.05,0.28) |  | 0.00  (0.00,0.00) | 0.00  (0.00,0.00) | 0.07  (-0.01,0.16) | 0.04  (-0.02,0.10) | 0.18  (0.06,0.30) |
| T2D+CHD | 0.00  (0.00,0.00) | 0.00  (0.00,0.00) | 0.05  (-0.02,0.12) | 0.04  (-0.02,0.10) | 0.04  (-0.02,0.1) |  | 0.03  (-0.03,0.09) | 0.00  (0.00,0.00) | 0.07  (-0.01,0.16) | 0.10  (0.01,0.19) | 0.20  (0.08,0.33) |
| Stroke+CHD | 0.03  (-0.03,0.09) | 0.00  (0.00,0.00) | 0.02  (-0.02,0.07) | 0.00  (0.00,0.00) | 0.12  (0.02,0.22) |  | 0.00  (0.00,0.00) | 0.00  (0.00,0.00) | 0.00  (0.00,0.00) | 0.02  (-0.02,0.06) | 0.06  (-0.01,0.13) |
| CMM with 2 diseases | 2.66  (2.1,3.22) | 4.31  (3.06,5.55) | 7.23  (6.44,8.02) | 6.31  (5.52,7.10) | 9.58  (8.62,10.54) |  | 3.46  (2.82,4.09) | 6.06  (4.85,7.27) | 7.15  (6.36,7.94) | 5.81  (5.15,6.46) | 8.02  (7.26,8.78) |
| HBP+T2D+Stroke | 0.03  (-0.03,0.09) | 0.27  (0.01,0.53) | 0.27  (0.11,0.43) | 0.14  (0.04,0.25) | 0.26  (0.12,0.41) |  | 0.09  (-0.01,0.20) | 0.20  (-0.03,0.43) | 0.22  (0.08,0.36) | 0.27  (0.12,0.41) | 0.22  (0.09,0.36) |
| HBP+T2D+CHD | 0.00  (0.00,0.00) | 0.07  (-0.06,0.20) | 0.05  (-0.02,0.12) | 0.16  (0.05,0.28) | 0.22  (0.09,0.36) |  | 0.03  (-0.03,0.09) | 0.07  (-0.06,0.20) | 0.07  (-0.01,0.16) | 0.29  (0.14,0.44) | 0.31  (0.15,0.46) |
| Stroke+CHD+HBP | 0.00  (0.00,0.00) | 0.00  (0.00,0.00) | 0.05  (-0.02,0.12) | 0.10  (0.01,0.19) | 0.04  (-0.02,0.10) |  | 0.06  (-0.02,0.15) | 0.07  (-0.06,0.20) | 0.07  (-0.01,0.16) | 0.10  (0.01,0.19) | 0.14  (0.04,0.25) |
| Stroke+CHD+T2D | 0.00  (0.00,0.00) | 0.00  (0.00,0.00) | 0.00  (0.00,0.00) | 0.00  (0.00,0.00) | 0.02  (-0.02,0.06) |  | 0.00  (0.00,0.00) | 0.00  (0.00,0.00) | 0.00  (0.00,0.00) | 0.00  (0.00,0.00) | 0.02  (-0.02,0.06) |
| CMM with 3 diseases | 0.03  (-0.03,0.09) | 0.49  (0.06,0.92) | 0.36  (0.18,0.54) | 0.55  (0.31,0.79) | 0.74  (0.46,1.02) |  | 0.19  (0.04,0.34) | 0.33  (0.04,0.62) | 0.37  (0.18,0.55) | 0.65  (0.43,0.88) | 0.69  (0.46,0.92) |
| CMM≥2 diseases | 2.69  (2.13,3.25) | 4.79  (3.48,6.10) | 7.59  (6.79,8.40) | 6.86  (6.04,7.68) | 10.38  (9.39,11.37) |  | 3.68  (3.02,4.33) | 6.39  (5.15,7.63) | 7.52  (6.71,8.33) | 6.48  (5.79,7.17) | 8.76  (7.97,9.55) |

# Table S10 The association between CMM status and mortality

|  |  |  |  |
| --- | --- | --- | --- |
|  |  |  |  |
|  |  |  |  |
|  | |  |  |
|  |  |  |  |
|  |  |  |  |
|  |  |  |  |
|  |  |  |  |

|  | Without CMD | Single CMD | CMM |
| --- | --- | --- | --- |
| No. participants | 19405 | 10122 | 4717 |
| No. death  (%) | 330  (1.70%) | 536  (5.29%) | 681  (14.43%) |
| HR for all-cause mortality  (95% CI) | |  |  |
| Model 1 | REF | 1.48  (1.28,1.70) | 2.73  (2.37,3.14) |
| Model 2 | REF | 1.50  (1.30,1.72) | 2.69  (2.33,3.09) |
| Model 3 | REF | 1.49  (1.29,1.71) | 2.70  (2.35,3.11) |
| Model 4 | REF | 1.43  (1.23,1.65) | 2.53  (2.19,2.93) |
| Model 5 | REF | 1.48  (1.28,1.71) | 2.68  (2.32,3.09) |

HRs for all-cause mortality were estimated using 5 models.

Model 1: adjusted for sex and age.

Model 2: Model 1 + region, education, marital status, ethnicity, job.

Model 3: Model 2 + smoking status (previously smoked and never smoke), drinking status (previously drank and never drink), other chronic diseases (no and yes).

Model 4: Model 3 + exclude participants with baseline before 2012.

Model 5: Model 3 + exclude participants whose first cardiovascular disease (C) and first metabolic disease (M) were diagnosed within 30 days of each other.

# Table S11 The associations between the number of CMD and mortality

|  |  |  |  |  |
| --- | --- | --- | --- | --- |
|  |  |  |  |  |
|  |  |  |  |  |
|  | | |  |  |
|  |  |  |  |  |
|  |  |  |  |  |
|  |  |  |  |  |
|  |  |  |  |  |

|  | Without CMD | 1 CMD | 2 CMDs | ≥3 CMDs |
| --- | --- | --- | --- | --- |
| No. participants | 19405 | 10122 | 3672 | 1045 |
| No. death  (%) | 330  (1.70%) | 536  (5.29%) | 448  (12.20%) | 233  (22.30%) |
| HR for all-cause mortality  (95% CI) | | |  |  |
| Model 1 | REF | 1.41  (1.28,1.70) | 2.40  (2.07,2.80) | 3.73  (3.12,4.45) |
| Model 2 | REF | 1.50  (1.30,2.75) | 2.37  (2.03,2.75) | 3.70  (3.09,4.41) |
| Model 3 | REF | 1.49  (1.29,1.71) | 2.37  (2.04,2.75) | 3.65  (3.06,4.35) |
| Model 4 | REF | 1.43  (1.24,1.66) | 2.28  (1.95,2.66) | 3.29  (2.73,3.95) |
| Model 5 | REF | 1.49  (1.29,1.71) | 2.37  (2.04,2.75) | 3.65  (3.06,4.35) |

Hazard ratios (HRs) for all-cause mortality were estimated using 5 models.

Model 1: adjusted for sex and age.

Model 2: Model 1 +region, education, marital status, ethnicity, job.

Model 3: Model 2 + smoking status (previously smoked and never smoke), drinking status (previously drank and never drink), other chronic diseases (no and yes).

Model 4: Model 3 + exclude participants with baseline before 2012.

Model 5: Model 3 + exclude participants whose first cardiovascular disease (C) and first metabolic disease (M) were diagnosed within 30 days of each other.

# Table S12 The associations between different multimorbidity development trajectories and mortality

|  |  |  |  |  |
| --- | --- | --- | --- | --- |
|  |  |  |  |  |
|  |  |  |  |  |
|  | | |  |  |
|  |  |  |  |  |
|  |  |  |  |  |
|  |  |  |  |  |
|  |  |  |  |  |
|  |  |  |  |  |

|  | Baseline to M | Baseline to C | Baseline to M to C | Baseline to C to M |
| --- | --- | --- | --- | --- |
| No. participants | 792 | 912 | 192 | 167 |
| No. death  (%) | 47  (5.93%) | 105  (11.51%) | 24  (12.50%) | 37  (22.15%) |
| HR for all-cause mortality  (95% CI) | | |  |  |
| Model 1 | REF | 2.15  (1.52,3.05) | 1.91  (1.17,3.13) | 3.50  (2.27,5.41) |
| Model 2 | REF | 2.01  (1.41,2.85) | 1.80  (1.10,2.95) | 3.45  (2.21,5.34) |
| Model 3 | REF | 1.90  (1.34,2.71) | 1.77  (1.08,2.91) | 3.32  (2.13,5.16) |
| Model 4 | REF | 1.74  (1.16,2.60) | 1.46  (081,2.63) | 2.63  (1.59,4.33) |
| Model 5 | REF | 1.92  (1.35,2.74) | 1.86  (1.12,3.07) | 3.45  (2.19,5.43) |

Hazard ratios (HRs) for all-cause mortality were estimated using 5 models.

Model 1: adjusted for sex and age.

Model 2: Model 1 +region, education, marital status, ethnicity, job.

Model 3: Model 2 + smoking status+(previously smoked and never smoke), drinking status (previously drank and never drink)+other chronic diseases (no and yes).

Model 4: Model 3 + exclude participants with baseline before 2012.

Model 5: Model 3 + exclude participants whose first cardiovascular disease (C) and first metabolic disease (M) were diagnosed within 30 days of each other.

# Table S13 Multiplicity-adjusted P values for interaction tests between CMM status/trajectory groups and stratification variables on all-cause mortality

| Interaction | Raw P | Bonferroni P | FDR P |
| --- | --- | --- | --- |
| CMM status*Sex | 0.064 | 0.318 | 0.109 |
| CMM status*Age group | <0.001 | <0.001 | <0.001 |
| CMM status*Region | 0.065 | 0.327 | 0.109 |
| CMM status*Smoking status | 0.265 | 1.000 | 0.326 |
| CMM status*Drinking status | 0.641 | 1.000 | 0.614 |
| CMM trajectory*Sex | 0.265 | 1.000 | 0.620 |
| CMM trajectory *Age group | 0.003 | 0.017 | 0.017 |
| CMM trajectory *Region | 0.372 | 1.000 | 0.620 |
| CMM trajectory *Smoking status | 0.697 | 1.000 | 0.697 |
| CMM trajectory *Drinking status | 0.694 | 1.000 | 0.697 |


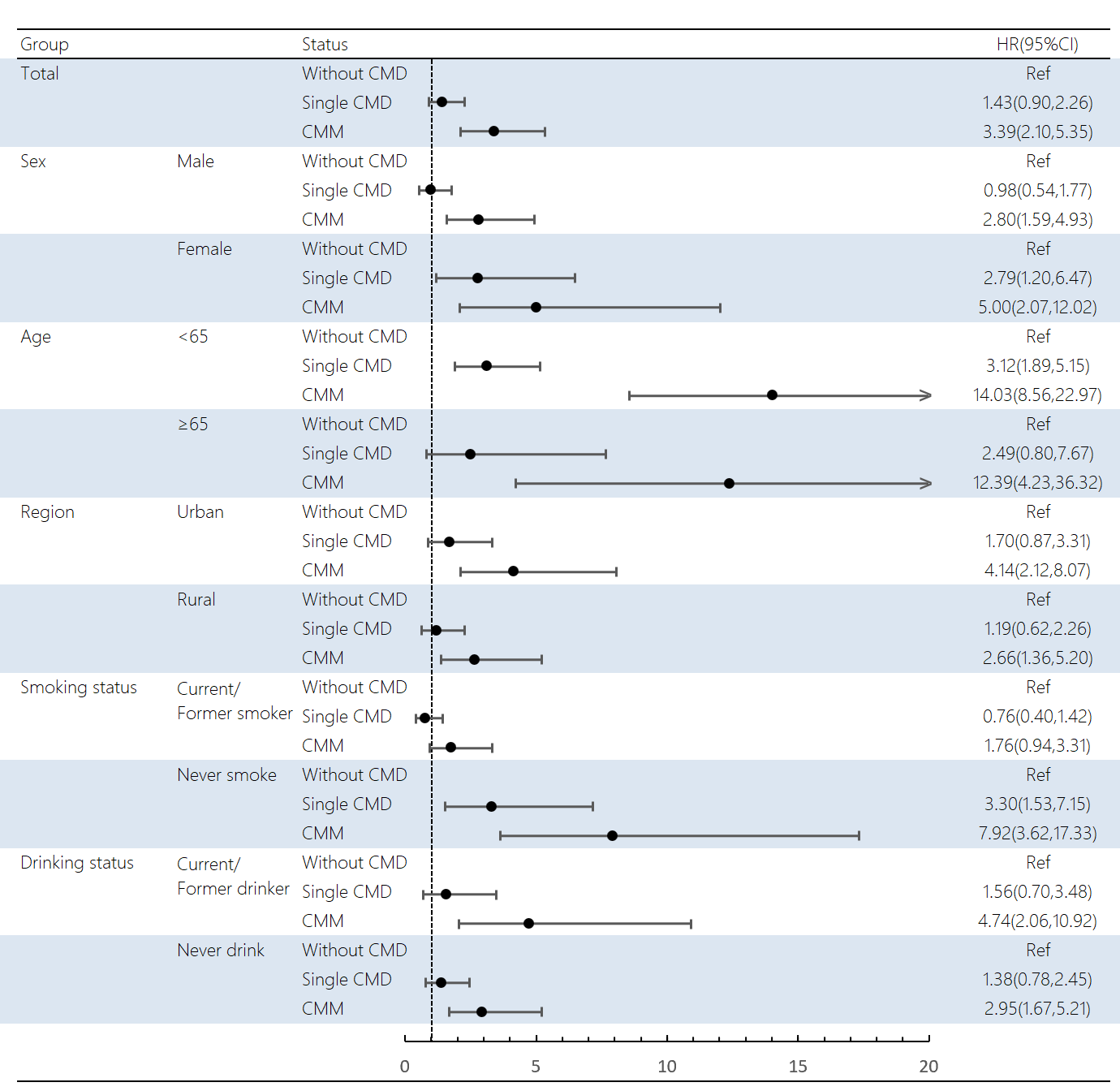


# Figure S3 Associations between CMM status and all‑cause mortality across subgroups (follow‑up ≥12 years).


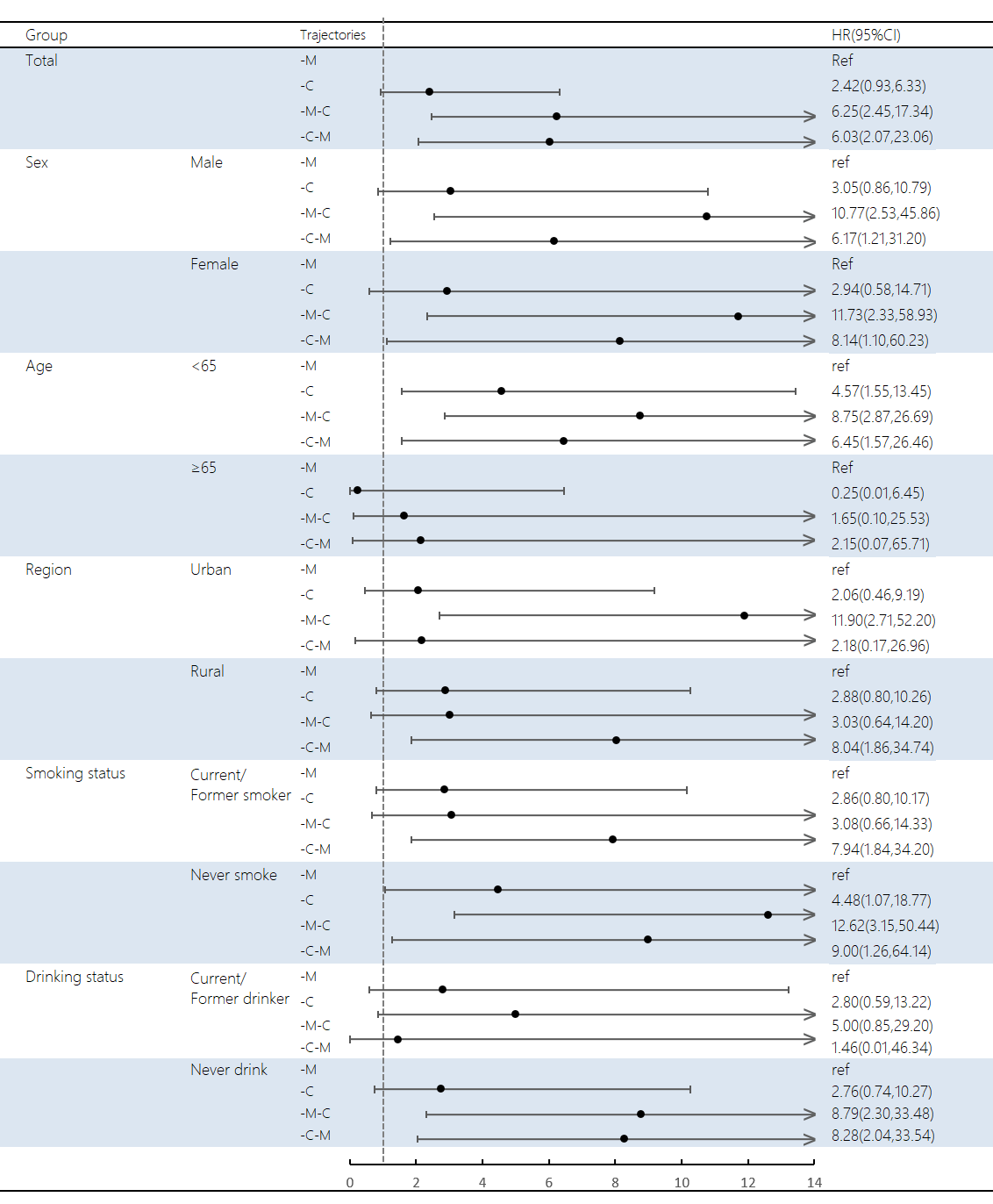


# Figure S4 Associations between CMM trajectories and all‑cause mortality across subgroups (follow‑up ≥12 years).

Table S14 The temporal trend of standardized CMD and CMM prevalence from 2007 to 2018 in Guangdong province

| CMD/CMM | Adjusted prevalence (%) of particpants aged≥18 | | | | |  | Adjusted prevalence (%) of particpants aged≥35 | | | | |  | Adjusted prevalence (%) of particpants aged≥40 | | | | |
| --- | --- | --- | --- | --- | --- | --- | --- | --- | --- | --- | --- | --- | --- | --- | --- | --- | --- |
|  | 2007 | 2010 | 2013 | 2015 | 2018 |  | 2007 | 2010 | 2013 | 2015 | 2018 |  | 2007 | 2010 | 2013 | 2015 | 2018 |
| HBP | 25.56 | 26.91 | 26.45 | 28.53 | 23.92 |  | 34.52 | 39.59 | 36.52 | 39.88 | 34.40 |  | 37.39 | 41.89 | 38.61 | 42.35 | 37.01 |
| T2D | 4.04 | 2.84 | 5.46 | 3.15 | 5.04 |  | 8.52 | 10.06 | 12.91 | 9.24 | 12.54 |  | 8.99 | 10.82 | 13.50 | 9.84 | 13.43 |
| CHD | 0.12 | 0.04 | 0.27 | 0.56 | 0.66 |  | 0.41 | 0.53 | 0.69 | 1.82 | 2.23 |  | 0.43 | 0.59 | 0.73 | 1.94 | 2.40 |
| Stroke | 0.08 | 0.00 | 0.17 | 0.41 | 0.47 |  | 0.65 | 1.19 | 1.24 | 1.83 | 2.33 |  | 0.70 | 1.22 | 1.34 | 2.01 | 2.51 |
| Single CMD | 29.80 | 29.79 | 32.35 | 32.65 | 30.10 |  | 34.40 | 35.81 | 34.55 | 37.16 | 31.97 |  | 36.82 | 37.61 | 36.08 | 39.22 | 34.07 |
| CMM | 4.31 | 5.86 | 7.36 | 6.50 | 8.75 |  | 4.52 | 7.00 | 7.80 | 6.75 | 8.64 |  | 5.01 | 7.59 | 8.38 | 7.44 | 9.39 |

Table S15 Sensitivity analysis restricting to different age ranges: associations of CMM status and trajectories with all-cause mortality among adults aged over 18, 35, and 40

| Exposure | Age over 18 HR(95%CI) | Age over 35 HR(95%CI) | Age over 40 HR(95%CI) |
| --- | --- | --- | --- |
| CMM status |  |  |  |
| Without CMD | 1.00(REF) | | |
| Single CMD | 1.49(1.29,1.71) | 1.48(1.28,1.70) | 1.47(1.27,1.69) |
| CMM | 2.70 (2.35,3.11) | 2.69(2.33,310) | 2.68(2.33,3.09) |
| CMM trajectory |  |  |  |
| H→M | 1.00(REF) | | |
| H→C | 1.90(1.34,2.71) | 1.90(1.34,2.71) | 1.90(1.31,2.75) |
| H→M→CMM | 1.77(1.08,2.91) | 1.89(1.14,3.13) | 1.83(1.09,3.04) |
| H→C→CMM | 3.32(2.13,5.16) | 3.37(2.13,5.30) | 3.19(2.01,5.05) |

# Table S16 E-values for the associations of CMD status and trajectory with all‑cause mortality, overall and by subgroups

| Exposure | HR(95%CI) | E-value (point estimate) | E-value (lower CI limit) |
| --- | --- | --- | --- |
| CMM status |  |  |  |
| Without CMD | 1.00(REF) | - | - |
| Single CMD | 1.49(1.29,1.71) | 2.38 | 1.96 |
| CMM | 2.70 (2.35,3.11) | 5.05 | 4.34 |
| CMM trajectory |  |  |  |
| H→M | 1.00(REF) | - | - |
| H→C | 1.90(1.34,2.71) | 3.22 | 2.01 |
| H→M→CMM | 1.89(1.14,3.13) | 2.95 | 1.38 |
| H→C→CMM | 3.37(2.13,5.30) | 6.09 | 3.69 |

E‑value: the minimum strength of association, on the risk ratio scale, that an unmeasured confounder would need to have with both the exposure and the outcome to fully explain away the observed association (point estimate) or to shift the lower confidence limit to the null (CI limit).
